# Compact target-aware stability-based feature selection for cross-dataset wearable activity monitoring

**DOI:** 10.1371/journal.pone.0359300

**Published:** 2026-10-07

**Authors:** Sarra Ayouni, Maha Sliti, Abeer Abdulaziz Alghanem, Fatimah Alhayan, Mohamed Maddeh

**Affiliations:** 1 Department of Information Systems, College of Computer and Information Sciences, Princess Nourah bint Abdulrahman University, Riyadh 11671, Saudi Arabia; 2 LR11TIC04, Communication Networks and Security Research Lab, and LR11TIC02, Green and Smart Communication Systems Research Lab, Higher School of Communication of Tunis (SUP’COM), University of Carthage, Ariana, Tunisia; 3 College of Applied Computer Science, King Saud University, Riyadh 11451, Saudi Arabia; Guizhou University of Finance and Economics, CHINA

## Abstract

Wearable activity-recognition models may lose reliability when transferred across participants, sensor placements, sampling rates, acquisition protocols, and activity definitions. This study evaluates a compact unsupervised target-aware feature-selection strategy for cross-dataset wearable activity monitoring under a pre-specified MHEALTH→PAMAP2 transfer protocol. Five-second wearable-signal windows were represented using 270 handcrafted inertial and magnetic descriptors. Source-domain mutual-information relevance was then combined with unlabeled source–target descriptor stability to select a compact set of 48 features, after which lightweight classifiers were trained using MHEALTH labels only. The original activities were harmonized into three coarse states: sedentary, active, and vigorous, while harmonized PAMAP2 target labels were withheld from model development and used only for final evaluation. Under leave-one-subject-out evaluation on MHEALTH, the Random Forest classifier achieved 0.9985 accuracy and 0.9984 macro-F1. In the primary cross-dataset experiment, the pre-specified stability-aware configuration achieved 0.9042 accuracy and 0.8753 macro-F1 on PAMAP2, compared with 0.8888 accuracy and 0.8436 macro-F1 for the full 270-feature representation and 0.8734 accuracy and 0.7940 macro-F1 for source-mutual-information-only selection. With all operating values frozen, reverse PAMAP2→MHEALTH transfer also ranked Stable-48 highest (accuracy 0.5811; macro-F1 0.4400), ahead of Source-MI-48 (0.5720; 0.4320) and Full-270 (0.5445; 0.4257), although Holm-adjusted participant-level contrasts were not significant. The compact configuration also achieved higher complete-window accuracy and macro-F1 than the tested shallow target-feature transformations and feature-level MLP adaptation baselines within the same handcrafted descriptor space. These findings indicate that jointly considering source-domain relevance and unlabeled cross-domain descriptor stability can improve compact cross-dataset wearable activity monitoring. The conclusions remain specific to the evaluated protocols and activity taxonomies and do not establish general superiority over raw-signal deep-learning methods or clinical readiness.

## 1 Introduction

Wearable sensing is widely used in mobile-health (mHealth) systems to monitor daily activity, functional behavior, rehabilitation progress, and health-related patterns outside laboratory settings [1–5]. Unlike episodic clinical or laboratory measurements, wearable devices can provide repeated observations during daily life and may support rehabilitation follow-up, chronic-condition monitoring, and smart-healthcare services [1,6–8]. For these applications, the objective is not only to recognize activity within a single dataset, but also to produce activity-related indicators that remain useful across users, devices, sensor placements, acquisition settings, and operating conditions.

Machine-learning methods are commonly used to convert wearable sensor streams into activity labels, functional-state indicators, or behavioral summaries [9,10]. Deep-learning models can perform well when large labeled datasets are available. However, lightweight feature-based models remain relevant for mHealth applications because they offer a practical balance between predictive performance, interpretability, computational cost, and deployment feasibility. These properties are particularly important when data are processed locally on smartphones, wearable gateways, or edge devices before compact descriptors or summarized outputs are transmitted to a remote platform.

A major limitation of wearable activity modeling is that strong performance on a single benchmark does not necessarily translate into reliable behavior in another sensing context. A model trained on one dataset may degrade when tested on data collected from different subjects, devices, sampling rates, body locations, activity protocols, or label definitions. This issue is central to wearable mHealth analytics because source-domain performance may overestimate real-world transferability. Cross-dataset evaluation is therefore needed to assess whether a model remains useful beyond the dataset on which it was trained.

This degradation is commonly associated with domain shift. In wearable sensing, domain shift may arise from inter-subject variability, hardware mismatch, sensor-placement differences, sampling-rate mismatch, acquisition-context changes, and inconsistent activity taxonomies. As a result, features that are discriminative in the source dataset may not preserve the same statistical behavior or decision relevance in the target dataset. Despite progress in wearable analytics, many studies still emphasize intra-dataset evaluation, while cross-dataset degradation, descriptor stability, class-wise reliability, calibration behavior, operating-point behavior, and subject-level variability remain less systematically examined [9,11]. Moreover, several domain-adaptation approaches rely on complex models or adaptation procedures that may be difficult to integrate into compact and interpretable mHealth pipelines [12,13].

Compact representation is also important for wearable mHealth systems. Transmitting raw multichannel windows can increase bandwidth use, storage demand, and cloud-side processing cost. A compact feature representation can reduce this burden if it preserves useful activity-monitoring performance under heterogeneous sensing conditions. This motivates approaches that jointly consider cross-dataset transfer, feature compactness, interpretability, and efficient inference, rather than optimizing source-domain accuracy alone.

In this study, we examine whether a compact subset of handcrafted wearable descriptors can support cross-dataset activity monitoring from *MHEALTH* to *PAMAP2*. The proposed pipeline includes preprocessing, window-based segmentation, handcrafted motion descriptors, source-domain relevance analysis, unlabeled target-domain descriptor-stability estimation, compact feature selection, and lightweight classification. The feature-selection step is target-aware because it uses unlabeled target-domain feature distributions to estimate descriptor stability. This setting assumes that an unlabeled batch of target-domain wearable windows is available before deployment or during a calibration phase. It is therefore transductive with respect to target-feature distributions, but remains unsupervised with respect to target labels. Target labels are reserved only for final evaluation and are not used for feature selection, model training, hyperparameter tuning, threshold selection, or operating-point selection. The resulting outputs are interpreted as wearable-derived activity indicators rather than diagnostic decisions, clinical assessments, or autonomous alarms. The same pipeline is also evaluated with *PAMAP2* as the labeled source dataset and *MHEALTH* as the unlabeled target dataset, using the operating values fixed for the primary analysis.

The primary evaluation uses *MHEALTH* as the source dataset and *PAMAP2* as the external target dataset. To improve comparability, the original activity labels are mapped into three coarse states: *sedentary*, *active*, and *vigorous*. These states provide a low-complexity operational abstraction for distinguishing posture-dominated behavior, routine movement, and more dynamic movement; they are not clinical diagnoses or validated metabolic-intensity bands. A complementary six-activity evaluation uses lying, sitting, standing, walking, stair climbing, and running to examine performance when biomechanically similar activities are separated. *UCI HAR* is included only as an exploratory extension and is not used to define the primary transfer protocol, select the main configuration, or support the principal performance claims. The evaluation covers source-domain performance, external transfer behavior, reverse-direction transfer, six-activity classification, feature-selection effects, sensitivity to stability-aware parameters, class-wise reliability, calibration-oriented metrics, operating-point behavior, subject-level variability, compactness, model-inference cost, software-side feature-extraction latency, and communication-payload estimates. Because target windows are grouped within subjects and are not statistically independent, window-level metrics are interpreted together with subject-level summaries.

The main contributions of this study are as follows:

- A cross-dataset wearable activity-monitoring protocol is defined using *MHEALTH* → *PAMAP2* as the primary transfer setting, with the original activity labels harmonized into the three coarse states *sedentary*, *active*, and *vigorous*.
- “‘Transfer-direction consistency is examined by reversing the dataset roles under the same fixed operating values, with *PAMAP2* as the labeled source and *MHEALTH* as the unlabeled target. A complementary six-activity evaluation examines whether the stability-aware selection behavior persists when lying, sitting, standing, walking, stair climbing, and running are represented as separate classes.
- An unsupervised target-aware stability-based feature-selection strategy is evaluated by combining source-domain discriminative relevance with label-free source–target descriptor-stability indicators. Within the proposed feature-selection procedure, unlabeled target-domain feature distributions are used only to estimate descriptor stability, whereas target labels remain inaccessible until final evaluation.
- The resulting compact representation is compared with full-feature transfer, source-only feature selection, and lightweight unsupervised target-feature transformations, including target z-score normalization, AdaBN-style normalization, mean–variance alignment, linear maximum mean discrepancy (MMD) mean matching, Transfer Component Analysis, and CORAL.
- Controlled feature-level neural adaptation baselines are included as supplementary transfer comparisons under the same target-label-free protocol. These models operate within the selected handcrafted descriptor space and are not presented as an exhaustive benchmark of raw-signal convolutional, recurrent, transformer-based, contrastive, or self-supervised activity-recognition architectures.
- The evaluation reports accuracy and macro-F1 together with class-wise behavior, calibration-oriented metrics, subject-level variability, leave-one-component-out stability-score ablations, sensitivity to *K* and α, sensitivity to alternative label harmonizations, model size, model-inference latency, software-side feature-extraction latency, and communication-payload estimates. “‘

Table 1 summarizes the methodological gap addressed in this study and shows how the proposed evaluation responds to limitations in wearable activity monitoring, domain adaptation, feature selection, and edge-compatible mHealth analytics.

**Table 1:** Summary of the methodological gap addressed in this study.

Research direction	Common limitation	Response in this study
Wearable activity monitoring	Many studies emphasize single-dataset performance.	Uses *MHEALTH* → *PAMAP2* as the primary cross-dataset transfer setting and interprets window-level results together with subject-level variability. Transfer-direction consistency is additionally examined under *PAMAP2* → *MHEALTH*, while a six-activity evaluation assesses finer-grained activity separation.
Domain adaptation for wearable sensing	Several approaches are complex or less interpretable.	Evaluates lightweight unsupervised transformations and compact target-aware stability-based feature selection under a no-target-label protocol.
Feature selection for wearable analytics	Source-only feature selection may retain dataset-specific descriptors.	Combines source-domain relevance with source–target descriptor stability using only unlabeled target-domain feature distributions.
Edge-compatible mHealth analytics	Compactness, latency, payload, and subject-level variability are not always jointly reported.	Reports feature count, model size, inference latency, feature-extraction latency, payload estimates, calibration-oriented metrics, and subject-level variability.

The rest of this paper is organized as follows. Section 2 reviews related work on wearable activity monitoring, cross-dataset reliability, lightweight adaptation, and compact wearable analytics. Section 3 presents the study framework and transfer setting. Section 4 describes the datasets, label harmonization, preprocessing, feature extraction, stability-aware feature selection, reverse-direction validation, six-activity evaluation, and evaluation protocol. Section 5 reports the experimental results, including primary and reverse-direction transfer performance, the six-activity evaluation, feature-level neural baselines, sensitivity analysis, reliability assessment, and latency analysis. Section 6 discusses the main findings, deployment implications, limitations, and future research directions. Section 7 concludes the paper.

## 2 Related work

### 2.1 Wearable activity monitoring and machine learning

Wearable sensing enables continuous observation of physical activity, functional behavior, and rehabilitation-related patterns outside controlled laboratory environments [1–4]. Such measurements support activity recognition, rehabilitation follow-up, behavioral monitoring, and patient-centered health analytics [5,7,8].

Machine-learning methods transform multichannel sensor streams into activity classes or broader behavioral indicators [9,10]. Although deep architectures can learn representations directly from raw signals, compact feature-based models remain relevant when interpretability, computational efficiency, and local execution are important [11]. Handcrafted window-level representations combined with lightweight classifiers therefore remain practical for resource-constrained wearable monitoring.

Recent work has also reinforced the value of interpretable signal-derived features. Msokar et al. [14] developed a multi-domain photoplethysmography feature-engineering framework for multi-class blood-pressure staging. Their study illustrates how structured descriptors can preserve feature traceability while supporting conventional machine-learning models. Although their modality and clinical task differ from activity recognition, the same feature-centric motivation underlies the present use of interpretable inertial and magnetic descriptors.

### 2.2 Cross-dataset reliability and domain shift

Strong performance within a single dataset does not necessarily imply reliable behavior in an external sensing environment. Cross-dataset degradation is commonly associated with domain shift [12,15], which in wearable sensing may arise from differences in participants, hardware, body placement, sampling frequency, acquisition protocol, activity execution, and label taxonomy [13,16]. Consequently, descriptors that are discriminative in the source dataset may change in scale, distribution, or predictive relevance after transfer.

External evaluation should therefore consider more than aggregate accuracy. Class-specific errors, calibration behavior, and inter-subject variability may remain hidden when multiple windows are pooled across participants. Cross-dataset studies should accordingly combine complete-window results with class-wise and subject-level analyses.

A further distinction is required between strict source-only transfer and unsupervised target-aware transfer. Source-only methods use no target-domain information before prediction, whereas target-aware methods may use unlabeled target observations to estimate statistics, align representations, or guide feature selection. The latter setting remains unsupervised with respect to target labels but is transductive with respect to the available target-feature distribution; it is therefore not equivalent to zero-target-data generalization.

### 2.3 Wearable domain adaptation and stability-aware feature selection

Domain-shift mitigation methods include normalization, distribution alignment, transferable representation learning, contrastive learning, and adversarial adaptation [12,13,15]. Recent wearable approaches increasingly learn transferable temporal representations directly from multivariate sensor sequences. Zhang et al. [17] proposed a contrastive domain-adaptation framework combining time-lag attention with teacher–student and class-conditional alignment mechanisms. Their work illustrates the potential of learned temporal invariance under wearable domain shift.

Such methods address a different operating point from compact handcrafted pipelines. They rely on trainable temporal encoders and adaptation losses, whereas feature-based approaches emphasize explicit descriptors, lower complexity, and easier inspection of the selected inputs. These directions are complementary: learned adaptation seeks expressive cross-domain representations, while compact feature selection seeks a transparent and computationally controlled representation.

Lighter alternatives based on unlabeled target data include target-statistics normalization, AdaBN-style normalization, mean–variance matching, CORAL, linear-MMD mean matching, and Transfer Component Analysis. These methods reduce marginal source–target mismatch without using target labels, but marginal alignment does not necessarily preserve class-conditional separability.

Stability-aware feature selection provides another alternative. A source-only ranking may retain descriptors that are discriminative during training but sensitive to dataset-specific conditions. Incorporating unlabeled source–target distributional information can instead favor descriptors that balance source relevance with cross-domain consistency. Because the target distribution contributes to the ranking, this procedure is appropriately described as unsupervised target-aware feature selection.

Unlike Zhang et al. [17], the present method does not learn a raw-signal temporal encoder, generate target pseudo-labels, or optimize a trainable alignment loss. It operates in an interpretable handcrafted descriptor space and uses unlabeled target distributions only to penalize descriptors with greater source–target instability.

### 2.4 Compact and edge-compatible wearable analytics

Local or edge-based wearable analytics can reduce continuous raw-signal transmission and support processing on smartphones, gateways, or resource-constrained devices [18–22]. Compact descriptors may reduce communication and storage requirements while preserving an interpretable input representation.

However, classifier inference time alone does not characterize the complete processing burden, because window construction and descriptor extraction may dominate the software-side cost. Deployment-oriented evaluation should therefore consider representation size, feature-extraction time, model-inference latency, communication payload, predictive reliability, and inter-subject variability together.

### 2.5 Position of this study

Prior wearable studies commonly emphasize single-dataset performance, source-only feature selection, shallow feature alignment, or learned raw-signal representations. Interpretable feature-engineering research demonstrates the value of transparent signal-derived descriptors [14], while recent domain-adaptation methods demonstrate the potential of learned temporal representations [17]. The present study examines a complementary question: whether a compact handcrafted representation can be selected by jointly considering source-domain discriminative relevance and unlabeled source–target descriptor stability.

The primary protocol uses *MHEALTH* as the labeled source dataset and *PAMAP2* as the external target dataset, while *UCI HAR* is retained only for exploratory analysis. The proposed selector is compared with the full descriptor space, source-mutual-information-only selection, shallow unsupervised target-feature transformations, and controlled feature-level Source-MLP, Deep CORAL-MLP, and DANN-MLP baselines.

The neural comparisons operate within the same handcrafted descriptor space and are not intended as an exhaustive benchmark of raw-signal convolutional, recurrent, transformer-based, contrastive, self-supervised, or foundation-style architectures. The contribution is instead a transparent target-label-free framework that jointly evaluates cross-dataset performance, feature-selection behavior, class-wise reliability, subject-level variability, compactness, latency, and communication payload.

## 3 Study framework and transfer setting

### 3.1 Overview

This study examines cross-dataset wearable activity monitoring under heterogeneous sensing conditions. A model trained on one dataset may degrade when applied to another because of differences in participants, devices, body placements, sampling rates, acquisition protocols, activity execution, and label taxonomies.

The proposed framework combines preprocessing, window-based segmentation, handcrafted descriptor extraction, source-domain relevance estimation, unlabeled source–target stability analysis, compact feature selection, and lightweight classification. Its objective is to retain descriptors that are discriminative in the source domain while remaining comparatively stable under external-domain shift.

The primary transfer protocol uses *MHEALTH* as the labeled source dataset and *PAMAP2* as the external target dataset. Their original activities are mapped into three coarse states: *sedentary*, *active*, and *vigorous*. These states are treated as wearable-derived activity indicators rather than clinical or diagnostic labels. The three-state mapping provides a low-complexity operational representation of posture-dominated behavior, routine movement, and more dynamic movement rather than a validated clinical or metabolic-intensity taxonomy. Transfer-direction consistency is also examined by using *PAMAP2* as the labeled source dataset and *MHEALTH* as the unlabeled target dataset under the same fixed operating values. A complementary finer-grained evaluation separates lying, sitting, standing, walking, stair climbing, and running to examine performance when biomechanically similar activities are no longer grouped into the same coarse state. *UCI HAR* is included only in an exploratory extension and is not used to define the primary protocol, select the main configuration, or support the principal performance claims.

Fig 1 summarizes the source-to-target pipeline. Unlabeled target descriptors may be used for stability estimation or unsupervised target-feature transformation, but harmonized target labels remain unavailable during feature selection, model training, transformation, hyperparameter specification, threshold specification, operating-point selection, and prediction generation. They are accessed only after predictions have been generated for final evaluation. The primary method is therefore unsupervised with respect to target labels but target-aware with respect to unlabeled target-feature distributions. The same processing, selection, training, and evaluation sequence is applied in the reverse direction after exchanging the source and target roles.

**Fig 1:**
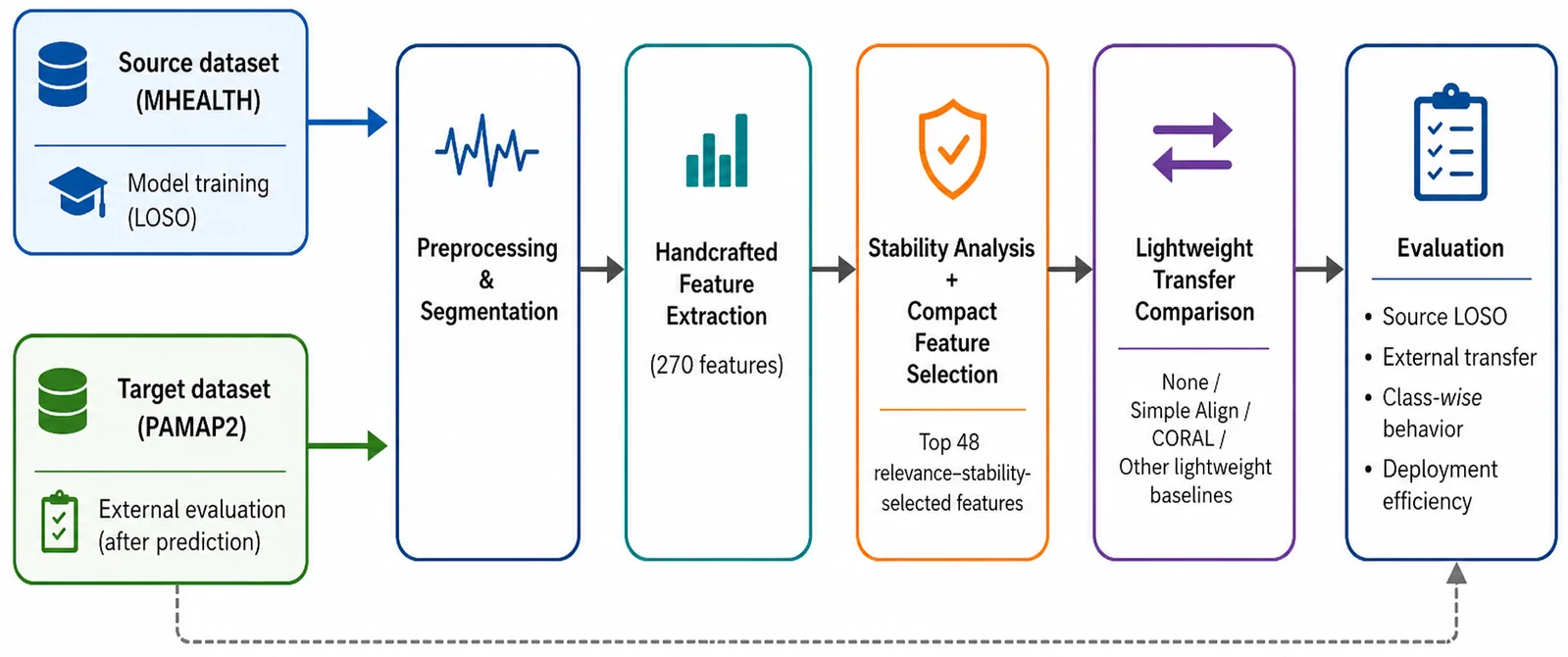
Source-to-target wearable activity-monitoring framework. A classifier is trained using labeled *MHEALTH* data and evaluated on the external *PAMAP2* dataset. After preprocessing, segmentation, and handcrafted feature extraction, descriptors are evaluated using source-domain relevance and unlabeled source–target stability. The selected compact representation is then used for source-model training and target prediction. Harmonized target labels remain inaccessible until final evaluation. The same pipeline is applied in the reverse-direction analysis after exchanging the source and target dataset roles.

### 3.2 Domain-shift formulation and transfer settings

Let the source and target domains be denoted by ${𝒟}_{S}$ and ${𝒟}_{T}$, respectively:


$$\begin{array}{cc} {𝒟}_{S} & ={\left\{({𝐱}_{i}^{S},y_{i}^{S})\right\}}_{i=1}^{N_{S}},\\ {𝒟}_{T} & ={\left\{({𝐱}_{j}^{T},y_{j}^{T})\right\}}_{j=1}^{N_{T}}. \end{array}$$ (1)

where $𝐱\in {\mathbb{R}}^{d}$ is a handcrafted descriptor vector and y∈{1,...,C} is the harmonized activity label. Here, *C* = 3, corresponding to *sedentary*, *active*, and *vigorous*. Source labels are available during model development, whereas harmonized target labels are sequestered until final evaluation. For the finer-grained evaluation, *C* = 6, corresponding to lying, sitting, standing, walking, stair climbing, and running.

The corresponding joint distributions are


$$\begin{array}{c} P_{S}(𝐱,y)\;\text{and}\;P_{T}(𝐱,y), \end{array}$$ (2)

and domain shift occurs when


$$\begin{array}{c} P_{S}(𝐱,y)\neq P_{T}(𝐱,y). \end{array}$$ (3)

In wearable sensing, this discrepancy may arise from differences in sensing hardware, body placement, sampling rate, acquisition conditions, activity composition, participant behavior, or feature–label relationships. Consequently, strong source-domain performance does not necessarily imply reliable target-domain behavior.

Three transfer settings are considered:

- *Strict external transfer*: the model is trained using the source dataset and evaluated on the target dataset without using target information before prediction.
- “‘*Unsupervised target-aware feature selection*: unlabeled target descriptors are used to estimate source–target stability and select a compact representation, without using target labels.
- *Unsupervised target-feature transformation*: source and unlabeled target descriptors are used to estimate a shallow normalization or alignment transformation before prediction. “‘

The term *no explicit target-feature transformation* means that no post-selection alignment method, such as mean–variance alignment, CORAL, linear-MMD mean matching, or TCA, is applied to the selected target descriptors. It does not exclude the use of unlabeled target distributions during stability-aware feature selection.

The target-aware settings assume that an unlabeled batch of target windows is available before deployment or during a calibration phase. They are therefore transductive with respect to the observed target-feature distribution and should not be interpreted as strict zero-target-data generalization. These target-label restrictions are applied unchanged in the primary transfer direction, the reverse transfer direction, and the six-activity evaluation.

### 3.3 Framework components and scope

The framework comprises three principal components. First, descriptor stability is characterized using normalized mean shift, distributional discrepancy, and source-relevance–target-dispersion rank consistency. Second, source-domain relevance and unlabeled cross-domain stability are combined to select a compact descriptor subset. Third, the selected representation is compared with full-feature transfer, source-only feature selection, shallow unsupervised target-feature transformations, and controlled feature-level neural baselines. Source mutual information, descriptor-stability quantities, and the selected feature subset are recomputed after reversing the source and target roles and after replacing the three-state mapping with the six-activity taxonomy.

The complete descriptor definitions, stability score, component ablations, target-feature transformations, and leakage-controlled evaluation procedure are provided in Subsections 4.6–4.8. Parameter-sensitivity analyses are used only to characterize robustness around the pre-specified primary configuration and are not treated as target-label-guided model selection.

Because overlapping target windows are clustered within participants, complete-window metrics are interpreted together with subject-level summaries. Higher aggregate accuracy or macro-F1 is therefore treated as descriptive target-window improvement rather than definitive evidence of subject-level superiority.

The framework is intended as a pre-deployment evaluation of wearable-derived activity indicators under cross-dataset shift. It does not replace clinical diagnosis, validated functional-assessment instruments, expert evaluation, or clinical decision-making. Its conclusions remain specific to the evaluated datasets, sensing conditions, transfer direction, and three-state harmonization. The reverse-direction and six-activity analyses examine the sensitivity of the observed behavior to transfer direction and label granularity but do not remove these scope limitations. Prospective validation, richer participant cohorts, embedded implementation, and independent clinical evaluation remain necessary before real-world healthcare deployment.

## 4 Materials and methods

This section describes the datasets, label harmonization, preprocessing, feature construction, baseline models, stability-aware feature selection, target-feature transformations, evaluation protocol, statistical analysis, and implementation. The study is unsupervised with respect to target labels but target-aware with respect to unlabeled target-feature distributions. Target-label access is restricted according to the leakage-control protocol defined in Subsection 4.8 and Algorithm 4.

### 4.1 Datasets and label harmonization

Three public wearable datasets are considered. *MHEALTH* is the labeled source dataset in the primary transfer protocol. It has a nominal sampling rate of 50 Hz and includes accelerometer, gyroscope, magnetometer, and ECG-related signals. *PAMAP2* is the external target dataset. It has a nominal sampling rate of 100 Hz and includes accelerometer, gyroscope, magnetometer, and heart-rate-related signals. ECG- and heart-rate-related channels are excluded so that the primary datasets are represented using their shared inertial and magnetic modalities. In the reverse-direction evaluation, the roles of these two datasets are exchanged while retaining the same shared modalities and descriptor construction.

*UCI HAR* is included only as an exploratory extension. It contains smartphone inertial measurements and differs from the primary datasets in sensor configuration, body placement, acquisition protocol, and original task definition. It is not used to define the primary transfer direction, select the primary feature-selection configuration, tune model parameters, or support the principal performance claims.

The two primary datasets are projected onto a common 12-channel inertial–magnetic representation. The retained accelerometer channels are acc_x, acc_y, acc_z, and acc_mag; the gyroscope channels are gyro_x, gyro_y, gyro_z, and gyro_mag; and the magnetometer channels are mag_x, mag_y, mag_z, and mag_mag.

Because the datasets do not share identical activity taxonomies, their original labels are mapped into three harmonized states: *sedentary*, *active*, and *vigorous*. This pre-specified mapping is an operational abstraction for controlled cross-dataset comparison rather than a unique biomechanical, physiological, or clinical taxonomy. Its practical purpose is to provide a low-complexity monitoring output that distinguishes the least dynamic posture-dominated periods, routine functional movement, and more dynamic activity bouts while reducing incompatibilities between the original dataset taxonomies. These states are not clinical diagnoses, validated functional-assessment categories, or metabolic-equivalent intensity bands.

Under consensus movement-behavior terminology, standing is a stationary but non-sedentary behavior [23]. Because standing is grouped with sitting and lying in the lowest-motion state, the term *sedentary* is used here only as an internal harmonization label and must not be interpreted as an estimate of consensus-defined sedentary behavior.

Activities grouped within the same state may differ in movement pattern, intensity, posture, body involvement, and biomechanical meaning. Activities with similar names may also be performed, sensed, or annotated differently across datasets. The resulting states are therefore interpreted as coarse wearable-derived activity indicators rather than interchangeable biomechanical classes or validated clinical activity levels.

The primary harmonization rules are reported in Table 2 and were fixed before model development and final target-label evaluation. Null, transient, and out-of-scope activities are removed according to the pre-specified filtering rules.

**Table 2:** Primary three-state label harmonization used for the main source-to-target transfer evaluation.

Dataset	Harmonized state	Original activities assigned to the state
*MHEALTH*	*Sedentary*	{1,2,3}: standing still, sitting and relaxing, lying down
*MHEALTH*	*Active*	{4,5,6,7,8,9}: walking, climbing stairs, waist bends forward, frontal elevation of arms, knees bending, cycling
*MHEALTH*	*Vigorous*	{10,11,12}: jogging, running, jump front and back
*PAMAP2*	*Sedentary*	{1,2,3,9,10,11}: lying, sitting, standing, watching TV, computer work, car driving
*PAMAP2*	*Active*	{4,6,7,12,13,16,17,18,19}: walking, cycling, Nordic walking, ascending stairs, descending stairs, vacuum cleaning, ironing, folding laundry, house cleaning
*PAMAP2*	*Vigorous*	{5,20,24}: running, playing soccer, rope jumping
*UCI HAR*	*Sedentary*	sitting, standing, laying
*UCI HAR*	*Active*	walking
*UCI HAR*	*Vigorous*	walking upstairs, walking downstairs

*Note:* The primary mapping is an operational cross-dataset abstraction rather than a unique biomechanical or clinical taxonomy. The *UCI HAR* mapping is used only in the exploratory analysis and does not contribute to selection of the primary configuration or the principal performance claims.

Two alternative mappings are evaluated in a post-hoc sensitivity analysis. In the *stairs-vigorous* variant, stair climbing in *MHEALTH* and ascending and descending stairs in *PAMAP2* are reassigned from *active* to *vigorous*. In the broader *exercise-vigorous* variant, cycling is additionally reassigned in *MHEALTH*, while cycling and Nordic walking are additionally reassigned in *PAMAP2*. For each mapping, source labels, source mutual information, feature selection, and RF training are recomputed using *K* = 48, α=0.60, and no explicit target-feature transformation. These variants characterize mapping dependence and do not redefine the primary taxonomy or configuration.

A complementary six-activity evaluation uses the common activity intersection comprising lying, sitting, standing, walking, stair climbing, and running. The corresponding *MHEALTH* activity identifiers are 3, 2, 1, 4, 5, and 11, respectively. The corresponding *PAMAP2* identifiers are 1, 2, 3, 4, {12,13}, and 5, respectively, with ascending and descending stairs combined into a single stair-climbing class because *MHEALTH* provides one stair-climbing label. All other activities are excluded from this evaluation. Source mutual information, descriptor-stability quantities, the selected feature subset, and RF training are recomputed under the six-class labels using *K* = 48, α=0.60, the fixed stability weights, and no explicit target-feature transformation. The mapped target labels are not used for feature standardization, stability estimation, feature selection, model fitting, or prediction generation.

The retained class distributions under the primary mapping are reported in Table 3. The mapping-sensitivity results are presented in Subsection 5.5 and Table 11.

**Table 3:** Retained window-level distribution under the primary three-state harmonization.

Dataset	Sedentary	Active	Vigorous	Total retained	Subjects retained
*MHEALTH*	734	1,428	569	2,731	10
*PAMAP2*	2,265	4,912	581	7,758	9
*UCI HAR*	5,627	1,722	2,950	10,299	30

*Note:* Counts are reported after activity filtering, harmonization, and window-label construction under the primary mapping. For *MHEALTH* and *PAMAP2*, they correspond to 5-s windows with 50% overlap and majority-vote labeling. *UCI HAR* uses its pre-windowed exploratory representation.

### 4.2 Ethics statement

This study is a secondary analysis of de-identified, publicly available wearable-sensor datasets. No new participants were recruited, no new recordings were collected, and no identifiable private information was accessed. Additional institutional ethics approval was therefore not required for this secondary analysis. Participant recruitment, consent, and ethical approval for the original data collections remain the responsibility of the dataset providers.

### 4.3 Preprocessing and feature extraction

Identifiers, unused timestamps, orientation fields, non-shared physiological signals, and administrative variables are removed. Shared channels are then constructed from the tri-axial accelerometer, gyroscope, and magnetometer measurements.

Let


$$\begin{array}{c} \left(s_{x,r}^{\left(q\right)}(t),s_{y,r}^{\left(q\right)}(t),s_{z,r}^{\left(q\right)}(t)\right) \end{array}$$

denote the *r*-th tri-axial triplet of sensor family q∈{acc,gyro,mag}, where $R_{q}$ is the number of available triplets. The shared axis-wise channel is


$$\begin{array}{c} c_{a}^{\left(q\right)}(t)=\frac{1}{R_{q}}\sum_{r=1}^{R_{q}}s_{a,r}^{\left(q\right)}(t),\;a\in \{x,y,z\}. \end{array}$$ (4)

The corresponding magnitude channel is


$$\begin{array}{c} c_{mag}^{\left(q\right)}(t)=\frac{1}{R_{q}}\sum_{r=1}^{R_{q}}\sqrt{{\left(s_{x,r}^{\left(q\right)}(t)\right)}^{2}+{\left(s_{y,r}^{\left(q\right)}(t)\right)}^{2}+{\left(s_{z,r}^{\left(q\right)}(t)\right)}^{2}}. \end{array}$$ (5)

Each retained channel $s_{j}(t)$ is standardized as


$$\begin{array}{c} {\tilde{s}}_{j}(t)=\frac{s_{j}(t)-\mu_{j}}{\sigma_{j}}, \end{array}$$ (6)

where $\mu_{j}$ and $\sigma_{j}$ are estimated without using target labels. During source-domain leave-one-subject-out evaluation, they are estimated from the training subjects in each fold. In the primary *MHEALTH* → *PAMAP2* experiment, they are estimated from *MHEALTH* and applied to both domains. In the reverse *PAMAP2* → *MHEALTH* experiment, the standardization parameters are re-estimated from *PAMAP2* and applied to both domains.

The resulting canonical signal is


$$\begin{array}{c} 𝐬(t)=[s_{1}(t),s_{2}(t),...,s_{m}(t)], \end{array}$$ (7)

with *m* = 12. If *T* is the signal duration, *L* is the window length, and ρ is the overlap ratio, the number of complete windows is


$$\begin{array}{c} N=\left\lfloor \frac{T-L}{L(1-\rho )}\right\rfloor +1. \end{array}$$ (8)

The main pipeline uses *L* = 5 s and ρ=0.50, corresponding to 250 samples for *MHEALTH* and 500 samples for *PAMAP2*. The same window duration and overlap are retained when the source and target roles are reversed and in the six-activity evaluation.

Each retained source window is assigned a harmonized label by majority voting:


$$\begin{array}{c} y_{i}=\text{arg}\max_{c\in \{1,2,3\}}\sum_{t\in W_{i}}𝕀\;\left(y(t)=c\right). \end{array}$$ (9)

For the six-activity evaluation, the same rule is applied with c∈{1,...,6}, corresponding to lying, sitting, standing, walking, stair climbing, and running. Windows are formed within contiguous activity segments, and segmentation is restarted at activity transitions, excluded activity intervals, or recording gaps so that a retained window does not span different activities. The activity-inclusion and boundary rules are fixed before feature processing.

Target reference labels are constructed under the corresponding fixed mapping only after prediction generation. Original target activity identifiers are used only to apply the pre-specified activity-inclusion and window-boundary rules. They are not used for standardization, imputation, descriptor-stability estimation, feature selection, classifier training, hyperparameter specification, operating-point selection, or prediction generation.

Each retained window is represented by 270 handcrafted descriptors from five families: statistical, shape/energy, temporal-dynamics, frequency-domain, and inter-channel dependency features:


$$\begin{array}{cc} {ℱ}_{stat} & =\{\mu ,\sigma ,\min,\max,med,IQR\},\\ {ℱ}_{se} & =\{RMS,MAD,E,ZCR\},\\ {ℱ}_{temp} & =\{\mu_{\Delta },\sigma_{\Delta },\overset{―}{\left|\Delta \right|}\},\\ {ℱ}_{freq} & =\{f_{dom},C_{spec},H_{spec},R_{LH}\}. \end{array}$$ (10)

Here, *E*, $f_{dom}$, $C_{spec}$, $H_{spec}$, and $R_{LH}$ denote energy, dominant frequency, spectral centroid, spectral entropy, and the low–high frequency-power ratio, respectively. Temporal descriptors are computed from the first-order difference sequence


$$\begin{array}{c} \Delta c(t)=c(t)-c(t-1). \end{array}$$

Inter-channel dependency is represented by the within-window Pearson correlation:


$$\begin{array}{c} r_{ab}=\frac{\sum_{t=1}^{L}\left(c_{a}(t)-{\bar{c}}_{a}\right)\left(c_{b}(t)-{\bar{c}}_{b}\right)}{\sqrt{\sum_{t=1}^{L}{\left(c_{a}(t)-{\bar{c}}_{a}\right)}^{2}}\sqrt{\sum_{t=1}^{L}{\left(c_{b}(t)-{\bar{c}}_{b}\right)}^{2}}}. \end{array}$$ (11)

Undefined descriptors are treated as non-finite and replaced using feature-wise medians estimated from the corresponding source training partition. The final feature dimension is


d=12(6+4+3+4)+(122)=270. (12)

The same 270-dimensional descriptor construction is used in the primary transfer, reverse transfer, and six-activity evaluation. Standardization, imputation, source-domain relevance, descriptor stability, and feature selection are re-estimated for the corresponding source–target direction and activity taxonomy.

Algorithm 1 summarizes the preprocessing, segmentation, label-handling, and feature-construction sequence. All data-dependent parameters are re-estimated within the corresponding source training partition.

**Algorithm 1 Preprocessing, segmentation, and handcrafted feature construction**

**Require:** Raw source recordings ${ℛ}^{S}$ with source labels ${𝐲}^{S}$; raw target recordings ${ℛ}^{T}$; window length *L* = 5 s; overlap ratio ρ=0.50

**Ensure:** Source feature matrix ${𝐗}^{S}$, source window labels ${𝐲}_{w}^{S}$, and unlabeled target feature matrix ${𝐗}^{T}$

 1: Retain the shared accelerometer, gyroscope, and magnetometer signals.

 2: Remove identifiers, unused timestamps, orientation fields, non-shared physiological channels, and administrative variables.

 3: **for all** sensor familie q∈{acc,gyro,mag} **do**

 4:  Construct the shared axis-wise channels using Equation (4).

 5:  Construct the magnitude channel using Equation (5).

 6: **end for**

 7: Form the 12-channel canonical source and target representations.

 8: Estimate standardization parameters from the relevant source training partition.

 9: Standardize the source and target channels using Equation (6).

10: Segment each recording into complete *L*-second windows with overlap ratio ρ.

11: For the six-activity evaluation, restart segmentation at activity transitions, excluded activity intervals, and recording gaps according to the pre-specified inclusion and boundary rules.

12: **for all** source and target windows $W_{i}$ **do**

13:  Extract statistical, shape/energy, temporal-dynamics, and frequency-domain descriptors.

14:  Compute all pairwise within-window Pearson correlations.

15:  Concatenate the descriptors into ${𝐱}_{i}\in {\mathbb{R}}^{270}$.

16: **end for**

17: Estimate feature-wise imputation medians from the relevant source training partition.

18: Replace non-finite source and target descriptor values using the source-derived medians.

19: Construct source-window labels using the pre-specified mapping and majority-voting rules.

20: Keep harmonized target reference labels unavailable during this procedure and use original target activity identifiers only to apply the fixed activity-inclusion and window-boundary rules.

21: **return** ${𝐗}^{S}$, ${𝐲}_{w}^{S}$, and ${𝐗}^{T}$.

### 4.4 Baseline classifiers

The candidate classifiers are Logistic Regression (LR), Decision Tree (DT), Random Forest (RF), Support Vector Machine (SVM), Multilayer Perceptron (MLP), and XGBoost (XGB). Their hyperparameters are fixed before target-domain evaluation.

LR uses max_iter = 2000 and solver = lbfgs. DT uses max_depth = 12 and min_samples_leaf = 5. RF uses n_estimators = 300 and min_samples_leaf = 2. SVM uses C = 3.0, kernel = rbf, and gamma = scale. MLP uses hidden layers (128,64) and max_iter = 250. XGB uses n_estimators = 300, max_depth = 6, learning_rate = 0.05, subsample = 0.9, and colsample_bytree = 0.9.

RF is used for the matched Full-270, Source-MI-48, and Stable-48 comparisons in the primary transfer, reverse transfer, and six-activity evaluation. For each transfer direction and activity taxonomy, the RF is retrained using the corresponding source data while retaining the same hyperparameters. The three feature representations are evaluated using identical source windows, target windows, and classifier settings within each experiment.

For the multiclass problem, the predicted label is


$$\begin{array}{c} {\hat{y}}_{i}=\text{arg}\max_{c\in \{1,...,C\}}p(c|{𝐱}_{i}), \end{array}$$ (13)

with *C* = 3 for the three-state evaluation and *C* = 6 for the fine-grained activity evaluation.

### 4.5 Feature-level neural adaptation baselines

Three supplementary feature-level neural baselines are evaluated under the same *MHEALTH* → *PAMAP2* protocol. Source-MLP is trained using labeled source data only. Deep CORAL-MLP combines source-supervised classification with covariance alignment between source and unlabeled target representations. DANN-MLP combines source-supervised classification with an adversarial domain-classification objective using source and unlabeled target representations.

All three models operate on the selected 48-dimensional handcrafted descriptor representation. Deep CORAL-MLP and DANN-MLP use unlabeled target descriptors only through their adaptation losses. Their architectures, optimization parameters, and adaptation-loss weights are fixed before final target-label evaluation. Results are reported as mean ± standard deviation over the fixed seeds 42, 43, and 44. Detailed architectural specifications, seed-level predictions, confusion matrices, and subject-level results are provided in the supplementary reproducibility package.

These models are controlled feature-level comparisons. They do not constitute exhaustive raw-signal convolutional, recurrent, transformer-based, contrastive, self-supervised, or foundation-style HAR benchmarks.

### 4.6 Stability-aware feature selection

Each descriptor $f_{k}$, k∈{1,...,d}, is evaluated according to its source-domain discriminative relevance and source–target distributional stability. Target feature values may be used, but target labels remain unavailable.

For each transfer direction, all source-domain relevance quantities, source–target stability indicators, joint scores, and selected feature indices are recomputed after assigning the corresponding datasets to ${𝒟}_{S}$ and ${𝒟}_{T}$. In the six-activity evaluation, these quantities are recomputed under the six-class source labels and the corresponding unlabeled target-feature distribution. Consequently, neither the reverse-direction Stable-48 subset nor the six-activity Stable-48 subset is inherited from the primary three-state *MHEALTH* → *PAMAP2* experiment.

The first stability indicator is the normalized source–target mean shift:


$$\begin{array}{c} \Delta_{k}^{\left(\mu \right)}=\frac{\left|\mu_{k}^{S}-\mu_{k}^{T}\right|}{\sigma_{k}^{S}+\sigma_{k}^{T}+\epsilon }, \end{array}$$ (14)

where ϵ>0 prevents numerical instability.

The second indicator measures the Jensen–Shannon divergence between the empirical source and target marginal distributions:


$$\begin{array}{c} D_{k}^{\left(dist\right)}=d_{JS}^{\;2}\left(p_{k}^{S},p_{k}^{T}\right), \end{array}$$ (15)

where the distributions are estimated using 32-bin histograms defined over the same pooled source–target range.

The third indicator compares source-domain relevance with unlabeled target-domain dispersion. Source relevance is measured using the mutual information $I_{k}^{S}$ between $f_{k}^{S}$ and the source labels. The target-side dispersion proxy is


$$\begin{array}{c} u_{k}^{T}=\log\left(1+\mathrm{Var}(f_{k}^{T})\right). \end{array}$$ (16)

The quantity $u_{k}^{T}$ is not interpreted as target-domain class relevance. High variance may reflect activity variation, sensor noise, participant heterogeneity, acquisition mismatch, or other nuisance factors. It is used only as a weak label-free dispersion and non-degeneracy proxy; source mutual information remains the only class-aware relevance quantity.

Let $r_{k}^{S}$ and $r_{k}^{T}$ be the descending ranks of $I_{k}^{S}$ and $u_{k}^{T}$, respectively. Their normalized discrepancy is


$$\begin{array}{c} \Delta_{k}^{\left(rank\right)}=\frac{\left|r_{k}^{S}-r_{k}^{T}\right|}{d-1}. \end{array}$$ (17)

This quantity is treated as a consistency regularizer rather than an estimate of target-label relevance.

Each stability indicator is independently min–max normalized:


$$\begin{array}{c} {\tilde{z}}_{k}=\frac{z_{k}-\min_{\ell }z_{\ell }}{\max_{\ell }z_{\ell }-\min_{\ell }z_{\ell }+\epsilon }. \end{array}$$ (18)

The stability penalty is


$$\begin{array}{c} S_{k}=w_{1}{\tilde{\Delta }}_{k}^{\left(\mu \right)}+w_{2}{\tilde{D}}_{k}^{\left(dist\right)}+w_{3}{\tilde{\Delta }}_{k}^{\left(rank\right)}, \end{array}$$ (19)

with


$$\begin{array}{c} w_{1}=0.40,\;w_{2}=0.35,\;w_{3}=0.25,\;w_{1}+w_{2}+w_{3}=1. \end{array}$$ (20)

Lower $S_{k}$ indicates greater source–target stability. Because the three component scores lie in [0,1] and their weights sum to one, no additional normalization of $S_{k}$ is applied.

After independently normalizing the source mutual-information values to obtain ${\tilde{I}}_{k}^{S}$, the joint relevance–stability score is


$$\begin{array}{c} Q_{k}=\alpha {\tilde{I}}_{k}^{S}-(1-\alpha )S_{k}. \end{array}$$ (21)

The first *K* descriptors in the descending $Q_{k}$ ranking are retained:


$$\begin{array}{c} {ℱ}_{K}^{*}=\mathrm{TopK}\left(\{Q_{k}{\}}_{k=1}^{d}\right),\;\left|{ℱ}_{K}^{*}\right|=K. \end{array}$$ (22)

The pre-specified primary configuration uses *K* = 48 and α=0.60. The value *K* = 48 retains 17.8% of the original 270-dimensional representation, corresponding to an 82.2% reduction, while preserving descriptors from all five predefined feature families. It was therefore adopted as a moderate compactness operating point.

Three matched feature representations are used in the comparative evaluations. Full-270 retains all candidate descriptors, Source-MI-48 retains the 48 descriptors with the highest source mutual information, and Stable-48 retains the 48 descriptors with the highest joint relevance–stability score $Q_{k}$. Source-MI-48 and Stable-48 are reconstructed independently for each transfer direction and activity taxonomy.

The value α=0.60 assigns 60% of the joint criterion to source relevance and 40% to cross-domain stability. This slightly relevance-dominant balance reflects that source mutual information is the only class-aware quantity, whereas the stability terms are label-free.

Neither *K* = 48 nor α=0.60 was selected through a grid search using *PAMAP2* labels. Both values were fixed before final target-label evaluation. The subsequent sensitivity analyses are therefore reported as post-hoc robustness assessments and are not used to redefine the primary configuration. The same values of *K*, α, and $\left(w_{1},w_{2},w_{3}\right)$ are retained without target-label-guided adjustment in the reverse transfer and six-activity evaluation.

A leave-one-component-out ablation removes each stability term individually and renormalizes the two remaining weights:


$$\begin{array}{c} S_{k}^{\left(-\mu \right)}=\frac{w_{2}}{w_{2}+w_{3}}{\tilde{D}}_{k}^{\left(dist\right)}+\frac{w_{3}}{w_{2}+w_{3}}{\tilde{\Delta }}_{k}^{\left(rank\right)}, \end{array}$$ (23)

$$\begin{array}{c} S_{k}^{\left(-dist\right)}=\frac{w_{1}}{w_{1}+w_{3}}{\tilde{\Delta }}_{k}^{\left(\mu \right)}+\frac{w_{3}}{w_{1}+w_{3}}{\tilde{\Delta }}_{k}^{\left(rank\right)}, \end{array}$$ (24)

$$\begin{array}{c} S_{k}^{\left(-rank\right)}=\frac{w_{1}}{w_{1}+w_{2}}{\tilde{\Delta }}_{k}^{\left(\mu \right)}+\frac{w_{2}}{w_{1}+w_{2}}{\tilde{D}}_{k}^{\left(dist\right)}. \end{array}$$ (25)

The corresponding weights are


$$\begin{array}{cc} (-\mu ):\; & (0,\;0.5833,\;0.4167),\\ (-dist):\; & (0.6154,\;0,\;0.3846),\\ (-rank):\; & (0.5333,\;0.4667,\;0). \end{array}$$ (26)

All ablation variants retain *K* = 48, α=0.60, RF, the same preprocessing procedure, and no explicit target-feature transformation. They are interpreted as post-hoc component diagnostics and do not redefine the pre-specified primary formulation.

Algorithm 2 summarizes the complete unsupervised target-aware stability-based feature-selection procedure.

**Algorithm 2 Unsupervised target-aware stability-based feature selection**

**Require:** Labeled source features $\left({𝐗}^{S},{𝐲}^{S}\right)$; unlabeled target features ${𝐗}^{T}$; stability weights $\left(w_{1},w_{2},w_{3}\right)$; trade-off parameter α; retained feature count *K*

**Ensure:** Selected feature-index set ${ℱ}_{K}^{*}$

 1: Let *d* denote the number of candidate descriptors.

 2: **for** *k*=1 to *d* **do**

 3:  Compute source mutual information $I_{k}^{S}$.

 4:  Compute $\Delta_{k}^{\left(\mu \right)}$ using Equation (14).

 5:  Estimate pooled-range, 32-bin source and target histograms.

 6:  Compute $D_{k}^{\left(dist\right)}$ using Equation (15).

 7:  Compute $u_{k}^{T}$ using Equation (16).

 8: **end for**

 9: Rank $I_{k}^{S}$ and $u_{k}^{T}$ to obtain $r_{k}^{S}$ and $r_{k}^{T}$.

10: Compute $\Delta_{k}^{\left(rank\right)}$ for all descriptors.

11: Independently normalize the three stability indicators.

12: Compute $S_{k}$ using Equation (19).

13: Normalize $\{I_{k}^{S}{\}}_{k=1}^{d}$.

14: Compute $Q_{k}$ using Equation (21).

15: Rank descriptors by descending $Q_{k}$.

16: Retain the first *K* descriptors as ${ℱ}_{K}^{*}$.

17: **return** ${ℱ}_{K}^{*}$.

### 4.7 Unsupervised target-feature transformations

Let ${𝐙}^{S}\in {\mathbb{R}}^{N_{S}\times K}$ and ${𝐙}^{T}\in {\mathbb{R}}^{N_{T}\times K}$ denote the selected source and target feature matrices. The evaluated modes are no explicit transformation, target z-score normalization, AdaBN-style normalization, mean–variance alignment, CORAL covariance alignment, linear-MMD mean matching, and Transfer Component Analysis (TCA). Their parameters are estimated from source and unlabeled target descriptors and fixed before final target-label evaluation.

Under the no-transformation mode,


$$\begin{array}{c} {\tilde{𝐙}}^{S}={𝐙}^{S},\;{\tilde{𝐙}}^{T}={𝐙}^{T}. \end{array}$$ (27)

Target z-score normalization uses


$$\begin{array}{c} {\tilde{𝐳}}^{T}=\frac{{𝐳}^{T}-\mu^{T}}{\sigma^{T}+\epsilon }. \end{array}$$ (28)

AdaBN-style normalization replaces prediction-time normalization statistics with those estimated from the unlabeled target batch while keeping the source-trained model parameters fixed.

Mean–variance alignment maps target descriptors to the source first- and second-order statistics:


$$\begin{array}{c} {\tilde{𝐳}}^{T}=\left(\frac{{𝐳}^{T}-\mu^{T}}{\sigma^{T}+\epsilon }\right)⊙\sigma^{S}+\mu^{S}. \end{array}$$ (29)

Linear-MMD mean matching shifts the target mean to the source mean:


$$\begin{array}{c} {\tilde{𝐳}}^{T}={𝐳}^{T}-\mu^{T}+\mu^{S}. \end{array}$$ (30)

CORAL aligns the target covariance with the source covariance:


$$\begin{array}{c} {\tilde{𝐙}}^{T}=\left({𝐙}^{T}-1_{N_{T}}(\mu^{T}{)}^{⊤}\right){\left({𝐂}^{T}+\lambda 𝐈\right)}^{-1/2}{\left({𝐂}^{S}+\lambda 𝐈\right)}^{1/2}+1_{N_{T}}(\mu^{S}{)}^{⊤}. \end{array}$$ (31)

For TCA, a common projection 𝐏 is estimated from source and unlabeled target descriptors:


$$\begin{array}{c} {\tilde{𝐙}}^{S}={𝐙}^{S}𝐏,\;{\tilde{𝐙}}^{T}={𝐙}^{T}𝐏. \end{array}$$ (32)

Algorithm 3 summarizes the implementation sequence for the evaluated unsupervised target-feature transformations.

**Algorithm 3 Unsupervised target-feature transformation**

**Require:** Selected source features ${𝐙}^{S}$; unlabeled target features ${𝐙}^{T}$; transformation mode *m*

**Ensure:** Transformed source and target representations

1: **if** m=none **then**

2:  Retain both representations unchanged.

3: **else if** m=target-zscore **then**

4:  Estimate target mean and standard deviation.

5:  Apply Equation (28).

6: **else if** m=AdaBN **then**

7:  Replace prediction-time normalization statistics with target statistics.

8: **else if** m=align **then**

9:  Apply Equation (29).

10: **else if** m=MMD **then**

11:  Apply Equation (30).

12: **else if** m=CORAL **then**

13:  Estimate regularized source and target covariance matrices.

14:  Apply Equation (31).

15: **else if** m=TCA **then**

16:  Estimate the common projection from source and unlabeled target descriptors.

17:  Apply Equation (32).

18: **end if**

19: **return** the transformed representations.

Fixed transformation parameters and executable scripts are provided in the supplementary reproducibility package.

### 4.8 Evaluation protocol

The evaluation comprises source-domain benchmarking, the primary source-to-target experiment, reverse-direction transfer, a fine-grained six-activity evaluation, feature-selection and component ablations, parameter- and mapping-sensitivity analyses, target-feature transformations, feature-level neural baselines, and secondary reliability analyses. The pre-specified *MHEALTH* → *PAMAP2* experiment constitutes the principal analysis. All remaining experiments are used to characterize robustness, component contributions, mapping dependence, or alternative operating trade-offs and do not redefine the primary configuration.

Source-domain performance is evaluated using leave-one-subject-out cross-validation on *MHEALTH*. The classifier results reported in Table 4 use the complete 270-dimensional handcrafted descriptor representation. In each fold, imputation and standardization parameters are estimated exclusively from the *MHEALTH* training subjects and applied unchanged to the held-out subject. Model fitting is also performed using only the training subjects. No *PAMAP2* observations, target-domain distributions, or target-aware stability quantities are used in this source-domain benchmark. A separate source-only mutual-information analysis is conducted within the LOSO training folds to characterize feature-count sensitivity and is not used to generate the classifier results reported in Table 4.

**Table 4:** Source-domain benchmark performance on *MHEALTH* under leave-one-subject-out (LOSO) subject-independent evaluation. Values are averaged across LOSO folds; the final column reports the standard deviation of macro-F1 across folds.

Model	Accuracy	Macro-P	Macro-R	Macro-F1	Weighted-F1	Macro-F1 SD
RF	0.9985	0.9987	0.9981	0.9984	0.9985	0.0029
XGB	0.9978	0.9972	0.9983	0.9977	0.9978	0.0032
MLP	0.9971	0.9979	0.9966	0.9972	0.9971	0.0027
LR	0.9967	0.9976	0.9960	0.9968	0.9967	0.0026
SVM	0.9948	0.9964	0.9932	0.9947	0.9948	0.0043
DT	0.9871	0.9901	0.9833	0.9856	0.9868	0.0226

*Note:* Macro-P, Macro-R, and Macro-F1 denote macro-averaged precision, recall, and F1-score, respectively. All candidate classifiers use the complete 270-dimensional handcrafted descriptor representation. Imputation, standardization, and model fitting are estimated independently within each LOSO fold using only the *MHEALTH* training subjects and are applied unchanged to the held-out subject. No *PAMAP2* observations, target-domain distributions, or target-aware feature-selection quantities are used in this source-domain benchmark.

The primary transfer configuration uses stability-aware feature selection with *K* = 48, α=0.60, RF, and no explicit target-feature transformation. These operating values were fixed before final target-label evaluation and were not replaced on the basis of post-hoc sensitivity results.

The reverse-direction evaluation exchanges the dataset roles, using *PAMAP2* as the labeled source and *MHEALTH* as the unlabeled target. The values *K* = 48, α=0.60, the stability weights (0.40,0.35,0.25), the RF hyperparameters, the 5-s window duration, the 50% overlap, and the no-transformation setting remain unchanged. Source standardization, imputation, source mutual information, source–target stability quantities, feature rankings, selected subsets, and RF fitting are re-estimated after reversing the domain roles. Full-270, Source-MI-48, and Stable-48 are evaluated using the same source and target windows and the same RF configuration.

The fine-grained evaluation retains the primary *MHEALTH* → *PAMAP2* direction and uses the common activities lying, sitting, standing, walking, stair climbing, and running. PAMAP2 ascending and descending stairs are combined into the stair-climbing class. All other activities are excluded according to the fixed inclusion rules. Source labels, source mutual information, source–target stability quantities, feature rankings, selected subsets, and RF fitting are recomputed under the six-class taxonomy. Full-270, Source-MI-48, and Stable-48 are compared using the same frozen values of *K*, α, the stability weights, RF hyperparameters, window configuration, and no-transformation setting.

Unlabeled *PAMAP2* descriptors may be used only in explicitly target-aware operations, namely descriptor-stability estimation, target-feature transformation, Deep CORAL alignment, and DANN adaptation. For each evaluated configuration, *PAMAP2* labels remain inaccessible during preprocessing-parameter estimation, feature ranking, feature selection, model training, transformation estimation, adaptation-loss computation, hyperparameter specification, threshold selection, operating-point selection, and prediction generation. The predictions for that configuration are frozen before the corresponding target reference labels are constructed and used to compute the evaluation metrics.

The same target-label restrictions are applied to *MHEALTH* in the reverse-direction evaluation. Unlabeled *MHEALTH* descriptors may be used to compute the stability quantities, but its harmonized labels remain unavailable until the corresponding predictions have been frozen. In the six-activity evaluation, original target activity identifiers are used only to implement the fixed activity-inclusion and window-boundary rules described in Subsection 4.3. The resulting six-class target labels are not used for preprocessing, imputation, relevance or stability estimation, feature selection, RF fitting, parameter specification, or prediction generation.

The Source-MI-48 configuration evaluates whether dimensionality reduction based exclusively on source-domain relevance accounts for the observed transfer behavior. Component ablations remove one stability term at a time, with the remaining weights renormalized. The *K*- and α-sensitivity experiments are treated as post-hoc robustness analyses. In particular, the higher-performing *K* = 24 result is not used to replace the pre-specified *K* = 48 operating point.

For each alternative label mapping, the source labels, source-domain mutual information, selected descriptor subset, and RF model are recomputed. Mapping-specific target reference labels are constructed only during final evaluation. Target-feature transformations, feature-level neural baselines, the deployment-balanced configuration, and exploratory multi-dataset experiments are evaluated as separate secondary analyses and do not redefine the primary protocol.

Algorithm 4 summarizes the leakage-controlled evaluation sequence.

**Algorithm 4 Leakage-controlled source-domain and cross-dataset evaluation**

**Require:** Source recordings $\left({ℛ}^{S},{𝐲}_{orig}^{S}\right)$; target recordings ${ℛ}^{T}$; target labels ${𝐲}_{orig}^{T}$; mappings $h_{pri},h_{6},{ℋ}_{alt}$; fixed *K* = 48, α=0.60, stability weights, model settings, and neural seeds

**Ensrue:** Source-domain, primary-transfer, reverse-transfer, six-activity, secondary, and subject-level results

 1: Fix all primary operating values before target-label access.

 2: In every cross-dataset branch, keep harmonized target labels inaccessible until predictions are frozen; use original target activity identifiers only for the pre-specified inclusion and window-boundary rules.

  **Source-domain benchmark**

 3: **for all** *MHEALTH* subjects *s* **do**

 4:  Fit source-only preprocessing and each candidate classifier on all subjects except *s*; predict *s* using Full-270.

 5: **end for**

 6: Aggregate LOSO metrics and perform the source-only MI Top-*K* diagnostic within the LOSO training folds.

  **Primary *MHEALTH***→***PAMAP2* transfer**

 7: Construct descriptors under $h_{pri}$ using source-derived preprocessing.

 8: Compute Source-MI-48 and Stable-48 using source labels and unlabeled target descriptors.

 9: **for all** F∈{Full-270Source-MI-48,Stable-48} **do**

10:  Train RF on the corresponding source representation and store target predictions.

11: **end for**

  **Reverse *PAMAP2***→***MHEALTH* transfer**

12: Exchange source and target roles and recompute source-derived preprocessing, mutual information, stability quantities, rankings, and compact subsets.

13: **for all** F∈{Full-270Source-MI-48,Stable-48} **do**

14:  Retrain RF on *PAMAP2* and store *MHEALTH* predictions.

15: **end for**

  **Six-activity evaluation**

16: Restore *MHEALTH*→*PAMAP2*, apply *h*_6_, and retain lying, sitting, standing, walking, stair climbing, and running.

17: Recompute source-derived preprocessing, mutual information, stability quantities, rankings, and compact subsets under *h*_6_.

18: **for all** F∈{Full-270Source-MI-48,Stable-48} **do**

19:  Retrain RF under the six-class taxonomy and store target predictions.

20: **end for**

  **Secondary analyses**

21: **for all** fixed transformation, ablation, and sensitivity configurations **do**

22:  Estimate configuration-specific quantities without target labels, fit the model, and store target predictions.

23:  **end for**

24:  **for all** $h\in {ℋ}_{alt}$ **do**

25:  Reconstruct source labels, recompute source MI and ${ℱ}_{K,h}^{*}$, fit RF, and store target predictions.

26: **end for**

27: **for all** r∈{42,43,44} **do**

28:  Train Source-MLP, Deep CORAL-MLP, and DANN-MLP without target labels and store target predictions.

29: **end for**

  **Final evaluation**

30: **for all** stored configuration-specific prediction sets **do**

31:  Freeze predictions, construct the corresponding target reference labels, and compute the evaluation metrics.

32: **end for**

33: Compute complete-window, class-wise, participant-level, calibration, and bootstrap summaries.

34: **return** all evaluation results.

### 4.9 Evaluation metrics and statistical analysis

Performance is assessed using accuracy, balanced accuracy, macro-precision, macro-recall, macro-F1, weighted-F1, class-wise precision, recall, and F1-score, together with confusion matrices. Macro-F1 is emphasized because the harmonized classes are imbalanced and all three activity states are relevant for monitoring. Balanced accuracy is defined as the unweighted mean of the class-wise recalls:


$$\begin{array}{c} Balanced\;Accuracy=\frac{1}{C}\sum_{c=1}^{C}\frac{{TP}_{c}}{{TP}_{c}+{FN}_{c}}. \end{array}$$ (33)

For the six-activity evaluation, accuracy, balanced accuracy, macro-F1, and class-wise F1 are reported with a normalized 6×6 confusion matrix. Class-wise results are used to examine separation between biomechanically similar activities, particularly sitting versus standing and walking versus stair climbing.

For a metric *M*, transfer retention, absolute robustness gap, and relative degradation are defined as


$$\begin{array}{cccc} R_{M} & =\frac{M_{target}}{M_{source}},\;\Delta_{M} & =M_{source}-M_{target},\;G_{M} & =\left(1-\frac{M_{target}}{M_{source}}\right)\times 100\%. \end{array}$$ (34)

The stability of a selected descriptor subset ℱ is additionally summarized by its mean stability penalty:


$$\begin{array}{c} \overset{―}{S}(ℱ)=\frac{1}{\left|ℱ\right|}\sum_{k\in ℱ}S_{k}. \end{array}$$ (35)

Lower $\overset{―}{S}(ℱ)$ indicates that the selected descriptors have lower average source–target discrepancy under the stability criterion defined in Equation (19). For the six-activity evaluation, this quantity is reported for Source-MI-48 and Stable-48 to determine whether the stability-aware subset remains more stable under the finer-grained taxonomy.

Because overlapping windows from the same participant are not statistically independent, complete-window results are interpreted as descriptive performance indicators and are complemented by subject-level summaries.

For each target subject *s*, let $F_{1,s}^{\left(m\right)}$ denote the macro-F1 obtained by configuration *m*. Subject-level performance is summarized using the mean, standard deviation, minimum, and range across retained target subjects. For configurations *a* and *b*, the paired subject-level difference is defined as


$$\begin{array}{c} \Delta_{s}^{\left(a,b\right)}=F_{1,s}^{\left(a\right)}-F_{1,s}^{\left(b\right)}. \end{array}$$ (36)

A selected deployment-oriented paired comparison evaluates Stable-48 / align against the Full-270 / none baseline. The comparison is reported using the mean paired difference, a 95% subject-level bootstrap confidence interval, and a two-sided paired-bootstrap *p*-value. Given the limited number of retained target subjects, this analysis is interpreted descriptively and does not support definitive claims of subject-level superiority.

For the reverse-direction and six-activity evaluations, two paired comparisons are specified: Stable-48 versus Full-270 and Stable-48 versus Source-MI-48. Each comparison is summarized using the mean participant-level macro-F1 difference, the number of participants with positive differences, and a 95% participant-bootstrap confidence interval. A two-sided exact sign-flip test is applied to the paired participant-level differences. The two planned *p*-values within each evaluation are adjusted using the Holm procedure and reported as $p_{Holm}$. These participant-level analyses complement the complete-window metrics and are not replaced by window-level significance tests.

Subject-level bootstrap confidence intervals are estimated by resampling target subjects with replacement. Window-level confidence intervals are reported only as secondary descriptive estimates because overlapping windows within the same subject are not independent observations.

The deployment-balanced configuration is additionally evaluated using class-wise metrics, normalized confusion matrices, ordinal mean absolute error, multiclass Brier score, expected calibration error, subject-level heterogeneity, and a post-hoc vigorous-state threshold analysis. The threshold analysis characterizes the precision–recall trade-off and is not used for model selection or primary performance reporting.

### 4.10 Efficiency and implementation

Compactness is evaluated by comparing the 270-feature and 48-feature representations. Communication payload is estimated for raw-window, full-feature, compact-feature, and state-only transmission. These values are communication-size estimates and do not represent measured wireless energy consumption.

Model-inference latency is measured after descriptor extraction, whereas software-side feature-extraction latency is measured separately. The reported values do not include sensing, buffering, wireless transmission, device scheduling, or dashboard-side processing.

All experiments are implemented in Python within a unified preprocessing, feature-extraction, selection, classification, and evaluation workflow. Unless otherwise specified, the fixed random seed is 42. Runtime measurements are obtained on a CPU-based workstation under a consistent software environment.

The supplementary reproducibility package contains preprocessing and harmonization scripts, feature-extraction code, selected-feature lists, model configurations, random seeds, prediction outputs, mapping-sensitivity outputs, subject-level metrics, neural-baseline results, and the scripts used to generate the reported tables and figures.

## 5 Results

The primary *MHEALTH* → *PAMAP2* transfer experiment is evaluated together with secondary robustness, reliability, and exploratory analyses. For every reported configuration, target labels were excluded from preprocessing, feature selection, model fitting, adaptation, and prediction generation and were used only to evaluate the corresponding frozen predictions. Post-hoc analyses are interpreted as robustness and sensitivity assessments of the pre-specified primary configuration. Because overlapping windows from the same participant are not statistically independent, complete-window results are interpreted together with subject-level summaries.

### 5.1 Source-domain benchmark on *MHEALTH*

Table 4 shows near-ceiling subject-independent performance for all classifiers on *MHEALTH*. RF provides the highest overall result, whereas DT exhibits the largest fold-to-fold variability. These results confirm effective separation of the three harmonized states within the source dataset but do not establish external generalization.

### 5.2 Primary *MHEALTH*-to-*PAMAP2* transfer performance

Table 5 shows that the pre-specified Stable-48 / none configuration improves complete-window macro-F1 from 0.8436 for Full-270 / none to 0.8753, while reducing the representation from 270 to 48 descriptors. The source-only compact baseline does not provide the same benefit, indicating that compactness alone does not explain the observed improvement (Table 6).

**Table 5:** Representative *MHEALTH* → *PAMAP2* transfer configurations.

Configuration	Feat.	Acc.	Macro-F1	Vig. recall	Subj. gap	Role
Full-270 / none	270	0.8888	0.8436	0.6041	0.8284	Full-feature baseline.
Stable-48 / none	48	0.9042	0.8753	0.7143	0.5797	Main pre-specified compact setting.
Full-270 / align	270	0.8877	0.8384	0.9191	0.4546	High-recall, less compact setting.
Stable-48 / align	48	0.8519	0.7924	0.9191	0.3498	Deployment-balanced setting.
Stable-48 / TCA	48	0.8283	0.7378	0.5697	0.5118	TCA baseline.
Stable-48 / CORAL	48	0.6500	0.5884	0.7556	0.4386	CORAL baseline.
Stable-48 / MMD	48	0.6918	0.5642	0.8778	0.3407	MMD baseline.

*Note:* “none” denotes no explicit target-feature transformation, and “align” denotes simple mean–variance alignment. “Feat.” is the number of retained features, “Vig. recall” is vigorous-state recall, and “Subj. gap” is the target subject-level macro-F1 range. Complete-window results should be interpreted together with Table 6 and Subsection 5.10.

**Table 6:** Summary of subject-level macro-F1 values for representative *MHEALTH* → *PAMAP2* configurations.

Configuration	Mean	SD	Min	Range
Full-270 / none	0.7754	0.2590	0.0952	0.8284
Source-MI-48 / none	0.7348	0.2866	0.0000	0.9241
Stable-48 / none	0.7929	0.1964	0.3514	0.5797
Stable-48 / align	0.7079	0.1254	0.4894	0.3498

*Note:* Values are computed across the nine retained *PAMAP2* subjects. Full per-subject results are provided in Supporting Information.

Stable-48 / none remains target-aware because unlabeled *PAMAP2* descriptors contribute to stability estimation. Its higher complete-window performance is therefore interpreted as a descriptive transfer gain rather than evidence of zero-target-data generalization or definitive subject-level superiority.

The alignment-based configurations provide higher vigorous-state recall and lower subject-level dispersion but lower macro-F1. Stable-48 / align is therefore retained as a secondary deployment-oriented trade-off, whereas Stable-48 / none remains the primary configuration.

### 5.3 Supplementary feature-level neural adaptation baselines

Table 7 compares Source-MLP, Deep CORAL-MLP, and DANN-MLP within the same selected 48-dimensional handcrafted descriptor space. None of these models is trained directly on raw sensor windows.

**Table 7:** Controlled supplementary feature-level neural baselines under the *MHEALTH* → *PAMAP2* transfer protocol.

Configuration	Accuracy	Macro-F1	Vig. recall	Role
Source-MLP (feature-level)	0.5757 ± 0.0336	0.5638 ± 0.0224	0.9352 ± 0.0036	Source-only feature-level MLP.
Deep CORAL-MLP (feature-level)	0.6263 ± 0.0130	0.5990 ± 0.0105	0.9289 ± 0.0026	Feature-level covariance alignment.
DANN-MLP (feature-level)	**0.6578 ± 0.0502**	**0.6206 ± 0.0329**	0.9312 ± 0.0030	Feature-level adversarial adaptation.
Stable-48 / none	0.9042	0.8753	0.7143	Pre-specified stability-aware RF reference.

*Note:* Neural values are reported as mean ± standard deviation over three fixed seeds. The neural models operate on selected handcrafted descriptors and are not exhaustive raw-signal CNN, recurrent, transformer-based, contrastive, self-supervised, or foundation-style HAR benchmarks. Bold values identify the highest accuracy and macro-F1 among the three feature-level neural baselines.

DANN-MLP provides the highest mean accuracy and macro-F1 among the tested feature-level neural baselines, followed by Deep CORAL-MLP and Source-MLP. Thus, covariance-based and adversarial adaptation improve over the source-only MLP within this controlled descriptor-space comparison.

Stable-48 / none achieves higher complete-window accuracy and macro-F1 than all three feature-level MLP baselines. This finding is restricted to the implemented handcrafted representation and does not establish general superiority over raw-signal deep-learning or neural domain-adaptation methods.

Fig 2 summarizes this descriptor-space comparison.

**Fig 2:**
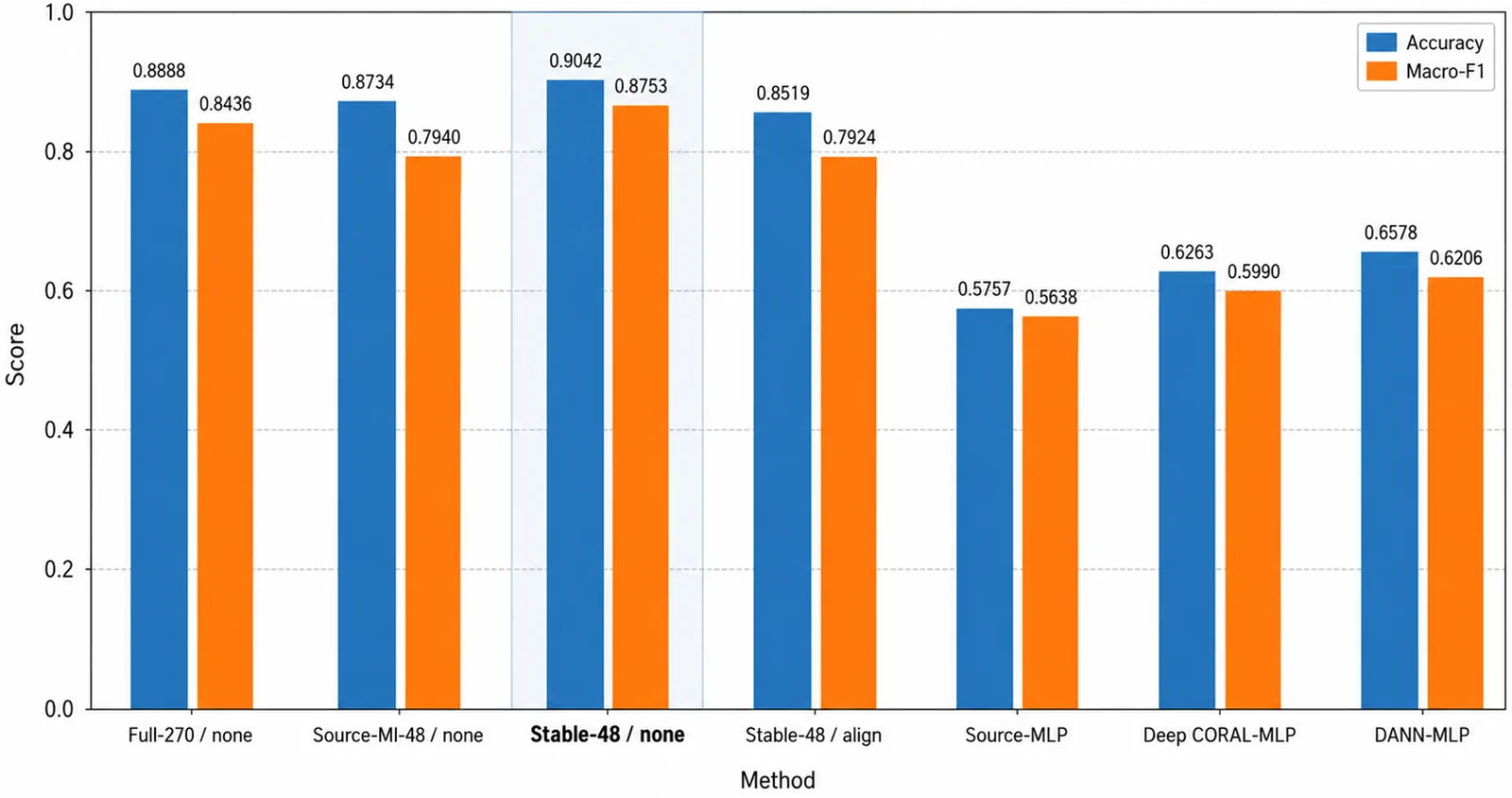
Complete-window cross-dataset performance under the *MHEALTH* → *PAMAP2* protocol. Stable-48 / none achieves higher accuracy and macro-F1 than the tested feature-level neural baselines within the common handcrafted descriptor space. The comparison does not establish subject-level or general raw-signal deep-learning superiority.

### 5.4 Feature-selection ablation and parameter sensitivity

Table 8 shows that Source-MI-48 / none performs below both Full-270 / none and Stable-48 / none. The advantage of Stable-48 is therefore associated with combining source relevance and unlabeled cross-domain stability rather than with dimensionality reduction alone.

**Table 8:** Feature-selection ablation under the *MHEALTH* → *PAMAP2* transfer protocol.

Feature setting	Dim.	Acc.	Macro-F1	Vig. recall	Interpretation
All features / none	270	0.8888	0.8436	0.6041	Full handcrafted representation.
Source-MI-48 / none	48	0.8734	0.7940	0.4492	Compact source-only selection.
**Stable-48 / none**	**48**	**0.9042**	**0.8753**	**0.7143**	**Pre-specified stability-aware configuration.**

*Note:* “none” denotes no explicit target-feature transformation. Source-MI-48 retains the 48 descriptors with the highest source-domain mutual information, whereas Stable-48 additionally incorporates unlabeled source–target stability.

The component ablation in Table 9 shows unequal contributions from the three stability terms. Removing mean shift produces the clearest deterioration and increases subject-level variability. Removing the rank-discrepancy term causes a smaller aggregate reduction but less favorable subject-level summaries, supporting its interpretation as a weak regularizer rather than a target-relevance measure.

**Table 9:** Leave-one-component-out ablation of the stability score under the primary *MHEALTH* → *PAMAP2* transfer protocol.

Stability setting	Accuracy	Macro-F1	Δ Macro-F1	Vig. recall	Subj. mean F1	Subj. range
Complete score	0.9042	0.8753	0.0000	0.7143	0.7929	0.5797
Without mean shift	0.8880	0.8632	−0.0121	0.7022	0.7953	0.7434
Without JSD	**0.9109**	**0.8857**	+0.0104	**0.7608**	**0.8012**	**0.5480**
Without rank discrepancy	0.8969	0.8712	−0.0041	0.7332	0.7723	0.6988

*Note:* Each ablation removes one component and renormalizes the remaining weights. All configurations use *K* = 48, α=0.60, RF, and no explicit target-feature transformation. The no-JSD result is post hoc and does not replace the primary configuration.

By contrast, the no-JSD variant improves all reported metrics. The ablation therefore does not demonstrate that all three terms are necessary for the primary transfer direction. The complete score is retained because it was pre-specified, while the no-JSD result is treated as a post-hoc diagnostic finding requiring independent validation.

The feature-count sensitivity analysis in Table 10 shows that configurations between *K* = 24 and *K* = 48 remain competitive. Although *K* = 24 achieves the highest post-hoc macro-F1 and a smaller subject-level range, it was identified after target-domain evaluation and is therefore not used to replace the pre-specified *K* = 48 setting.

**Table 10:** Post-hoc sensitivity analysis of the stability-aware feature-selection parameters under the *MHEALTH* → *PAMAP2* transfer protocol.

Setting	Accuracy	Macro-F1	Vigorous recall	Subject-level Macro-F1 range
*Feature-count sensitivity with fixed α=0.60*				
*K* = 24^†^	0.8948	**0.8859**	**0.8571**	**0.4696**
*K* = 36	**0.9042**	0.8761	0.7160	0.5784
K=48 ^*^	**0.9042**	0.8753	0.7143	0.5797
*K* = 64	0.8946	0.8616	0.6713	0.9226
*K* = 96	0.8827	0.8507	0.6575	0.8632
*Relevance–stability sensitivity with fixed K = 48*				
α=0.30	0.8900	0.8731	**0.9019**	**0.5041**
α=0.50	0.9037	**0.8766**	0.7160	0.5784
α=0.60 ^*^	**0.9042**	0.8753	0.7143	0.5797
α=0.70	0.8875	0.8633	0.7246	0.7447
α=0.90	0.8556	0.7621	0.3855	0.9131

*Note:* ^*^ denotes the pre-specified primary operating value. ^†^ denotes the post-hoc *K* = 24 configuration, which was evaluated only as part of the sensitivity analysis and was not used to replace the pre-specified *K* = 48 operating point. Bold values indicate the best result within each sensitivity block; tied accuracy values are both shown in bold. For the subject-level macro-F1 range, a smaller value indicates lower between-subject performance variability.

The α-sensitivity analysis favors intermediate relevance–stability balances. Values of 0.50 and 0.60 yield similar performance, whereas the strongly source-dominant α=0.90 setting substantially degrades macro-F1 and vigorous-state recall. The analysis supports a meaningful stability contribution but does not establish α=0.60 as universally optimal.

### 5.5 Label-harmonization sensitivity

Table 11 shows that the adopted activity harmonization materially affects both aggregate transfer performance and vigorous-state behavior. Relative to the primary mapping, the stairs-vigorous mapping reduces the macro-F1 of Stable-48 / none from 0.8753 to 0.6882 and its vigorous-state recall from 0.7143 to 0.3615. Under the broader exercise-vigorous mapping, its macro-F1 is 0.7058, whereas vigorous-state recall increases to 0.9021. Thus, reassigning activities to the vigorous state changes not only class prevalence but also the balance between aggregate classification performance and vigorous-state sensitivity.

**Table 11:** Post-hoc sensitivity to alternative three-state activity harmonizations under the *MHEALTH* → *PAMAP2* transfer protocol.

Activity mapping	Stable-48 / none accuracy	Stable-48 / none Macro-F1	Stable-48 / none vigorous recall	Source-MI-48 / none Macro-F1	Stable-48 / align Macro-F1
Primary mapping	**0.9042**	**0.8753**	0.7143	**0.7940**	0.7924
Stairs-vigorous	0.7448	0.6882	0.3615	0.6933	0.7257
Exercise-vigorous	0.7320	0.7058	**0.9021**	0.7140	**0.7514**

*Note:* In the stairs-vigorous mapping, stair activities are moved from *active* to *vigorous*. The exercise-vigorous mapping additionally moves cycling and Nordic walking as defined in Subsection 4.1. Feature selection and RF training are repeated for each mapping using *K* = 48 and α=0.60. Bold values denote the highest value within each column.

The configuration ordering also changes across mappings. Stable-48 / none achieves the highest macro-F1 among the reported compact settings under the primary mapping. In contrast, Stable-48 / align achieves higher macro-F1 than Stable-48 / none under both the stairs-vigorous mapping (0.7257 versus 0.6882) and the exercise-vigorous mapping (0.7514 versus 0.7058). The principal findings are therefore conditional on the pre-specified three-state taxonomy and should not be interpreted as invariant to alternative biomechanical or intensity-based harmonizations. Because the alternative mappings were evaluated post hoc, they characterize mapping sensitivity but do not redefine the primary taxonomy or operating configuration.

### 5.6 Feature-family stability and relevance

Fig 3 shows that correlation and frequency-domain descriptors are more stable on average, whereas statistical and temporal-dynamics descriptors provide stronger source-domain relevance but greater cross-domain variation. These complementary properties support their joint consideration in the stability-aware ranking.

**Fig 3:**
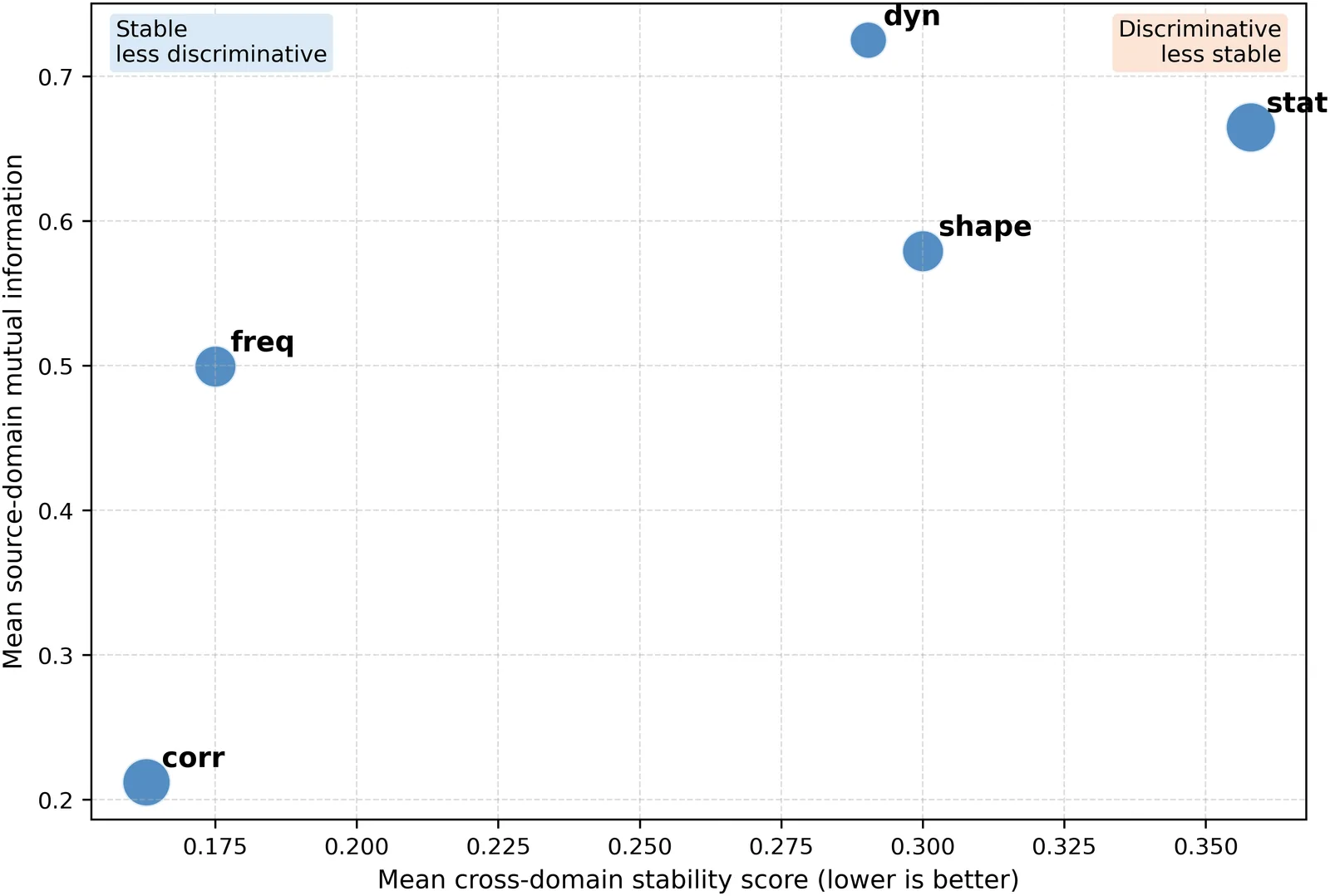
Trade-off between mean cross-domain stability score and mean source-domain discriminative relevance across feature families. Lower stability scores indicate greater source–target stability; bubble size reflects the number of descriptors in each family.

### 5.7 Compactness, payload reduction, and latency

Table 12 shows that transmitting the selected 48-feature vector instead of a raw 100 Hz window reduces the estimated payload from 24,000–192 bytes, corresponding to a 99.2% reduction. These values describe communication size and not measured energy consumption.

**Table 12:** Estimated per-window communication payload for representative transmission strategies.

Representation	Payload per window	Reduction vs. raw 100 Hz	Transmission time at 16 kbps
Raw 50 Hz window	12,000 bytes	50.0%	6.00 s
Raw 100 Hz window	24,000 bytes	0.0%	12.00 s
Full 270-feature vector	1080 bytes	95.5%	0.54 s
Selected 48-feature vector	192 bytes	99.2%	0.096 s
Predicted state only	4 bytes	99.98%	0.002 s

*Note:* Payloads assume 32-bit encoding for raw samples, feature values, and the predicted state. The values are communication-size estimates only.

Table 13 shows that software-side feature extraction dominates the measured processing cost, whereas classifier inference is negligible relative to the 5-s window duration. Fig 4 visualizes the payload comparison.

**Table 13:** Software-side latency profile of the proposed pipeline.

Stage	Time per window
Source windowing and feature extraction	27.63 ms
Target windowing and feature extraction	29.86 ms
Model inference after feature extraction	0.0215 ms

**Fig 4:**
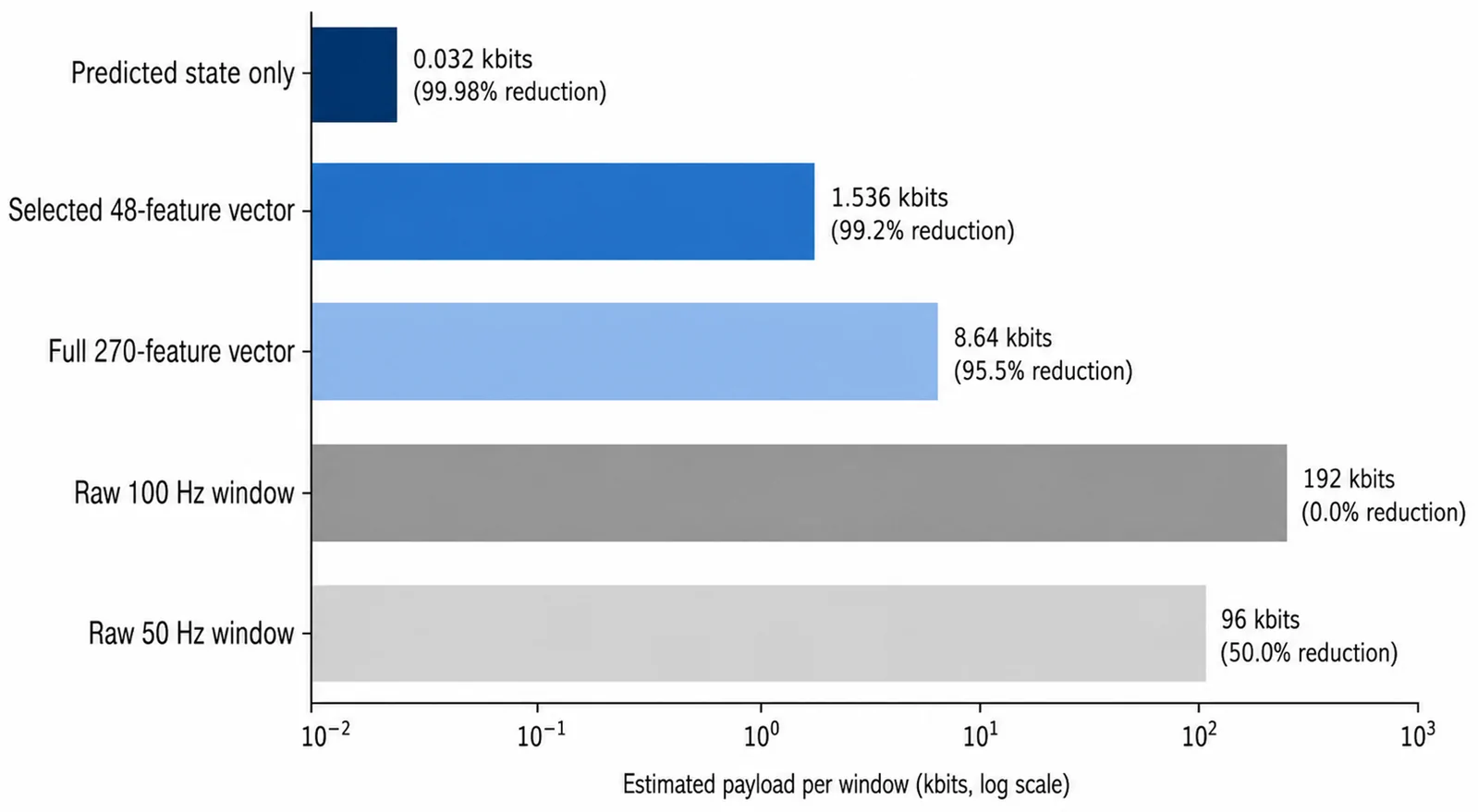
Payload comparison for raw-window, full-feature, compact-feature, and state-only transmission. The selected 48-feature representation reduces the estimated payload by approximately 99.2% relative to a raw 100 Hz window.

### 5.8 Reliability of the deployment-balanced configuration

Stable-48 / align is examined as a secondary deployment-oriented configuration because it combines compactness, high vigorous-state recall, reduced subject-level dispersion, and low model-inference latency. These advantages are obtained at the cost of lower macro-F1 than Stable-48 / none, so the configuration is interpreted as an operating trade-off rather than the best transfer setting.

Table 14 summarizes the predictive performance, calibration, compactness, model size, and inference latency of the selected deployment-balanced configuration on *PAMAP2*.

**Table 14:** Summary of the selected deployment-balanced configuration used for deeper validation on *PAMAP2.*

Metric	Value
Target Accuracy	0.8519
Target Macro-F1	0.7924
Target Weighted-F1	0.8629
Ordinal MAE	0.1498
Multiclass Brier Score	0.2202
Expected Calibration Error (ECE)	0.0549
Retained features	48
Model size	436.8 KB
Model-inference latency per target window	<0.03 ms

### 5.9 Class-wise behavior and vigorous-state operating point

The deployment-balanced configuration achieves high vigorous-state recall but lower precision, indicating that some active windows are classified as vigorous. It is therefore a sensitivity-oriented operating point that may produce excessive false positives if individual windows are treated as actionable events.

Figs 5 and 6 summarize the class-wise behavior. The post-hoc threshold analysis in Fig 7 confirms that increasing the vigorous threshold improves precision while reducing recall. This analysis is descriptive and is not used for model selection or primary reporting.

**Fig 5:**
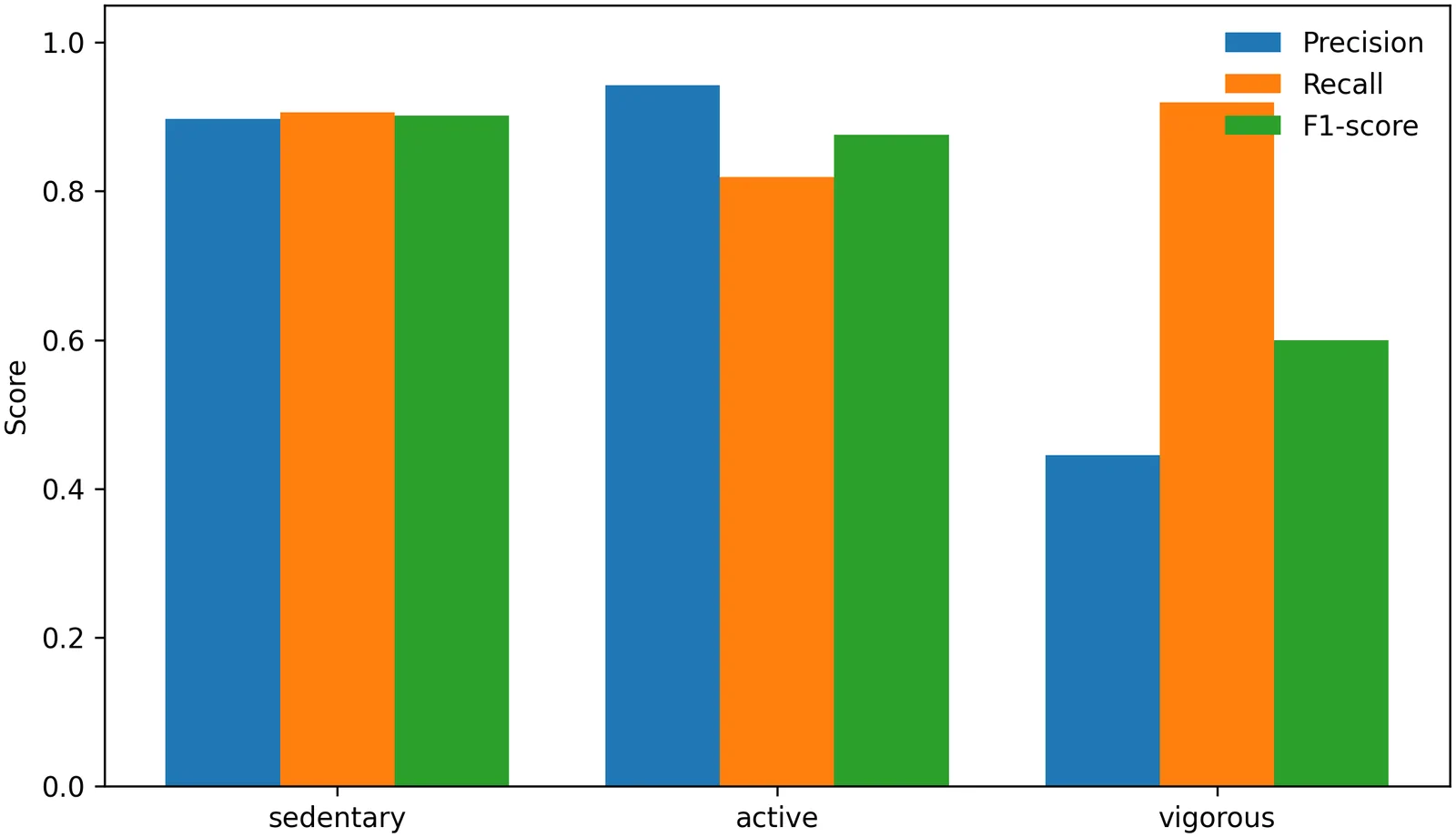
Class-wise precision, recall, and F1-score on *PAMAP2* for the deployment-balanced configuration.

**Fig 6:**
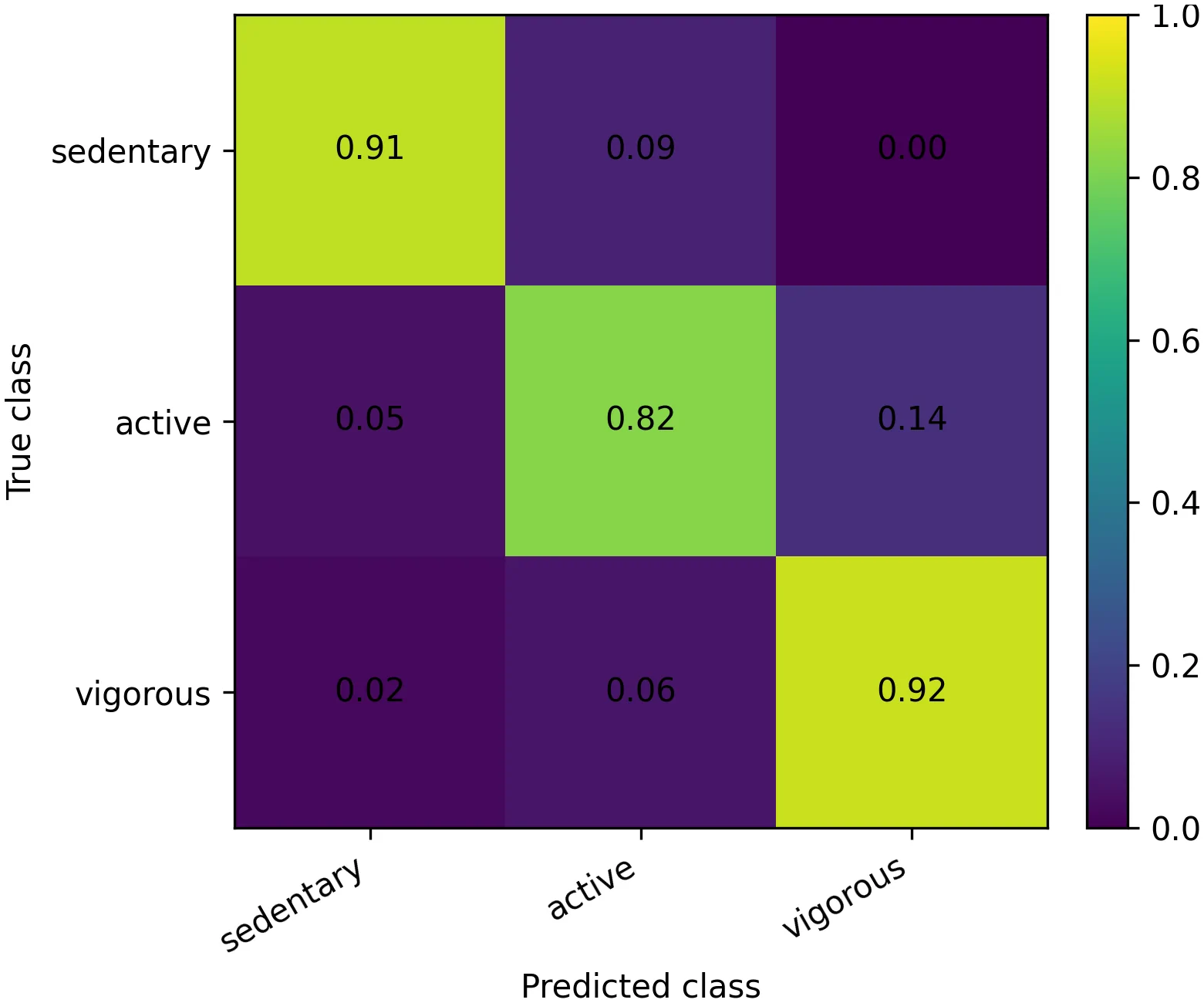
Normalized confusion matrix for the deployment-balanced configuration on *PAMAP2.* The dominant residual confusion occurs between active and vigorous states.

**Fig 7:**
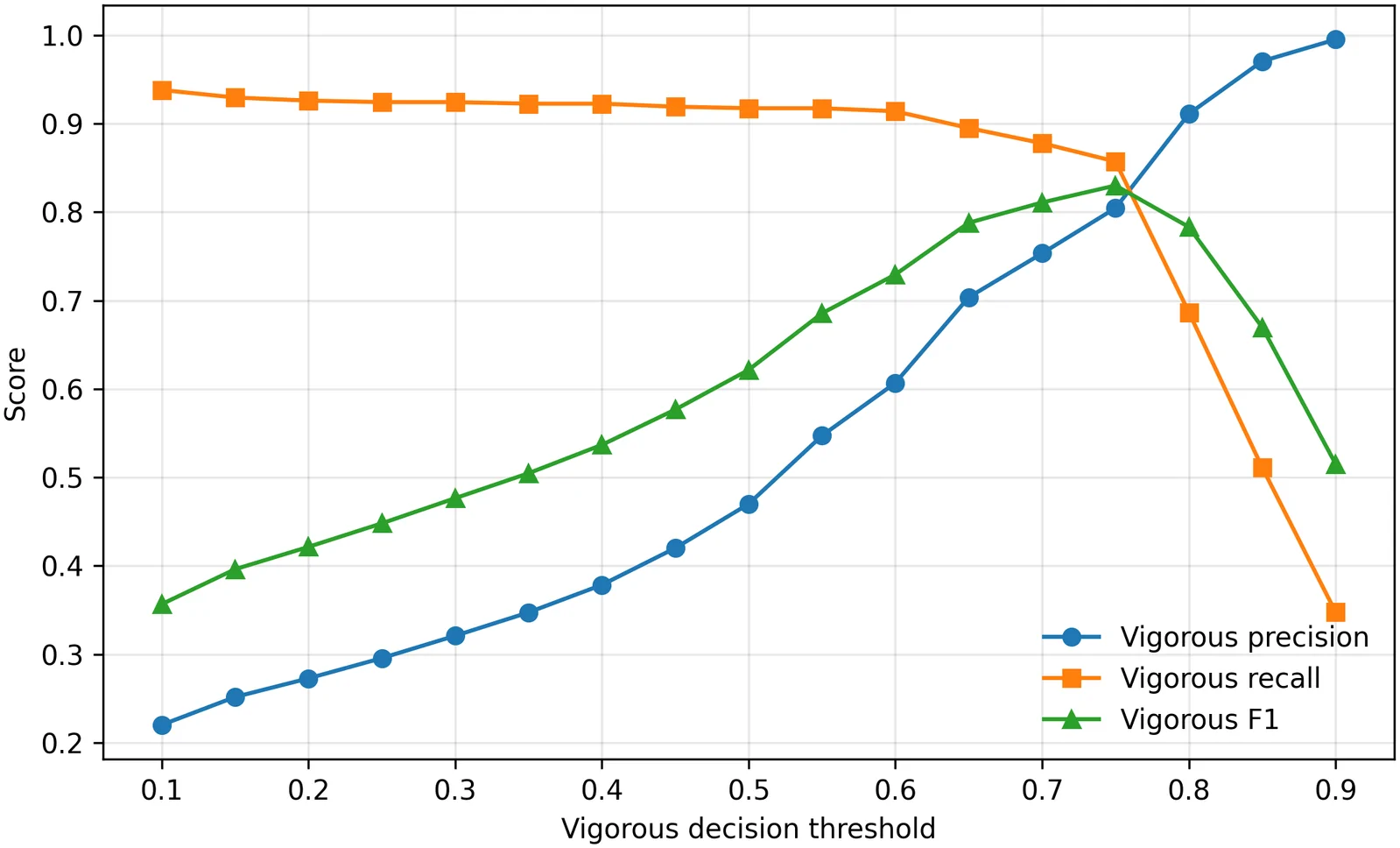
Post-hoc vigorous-state threshold sensitivity for the deployment-balanced configuration. Increasing the vigorous-state threshold improves precision while reducing recall. The analysis is not used for model selection or primary reporting.

### 5.10 Subject-level variability and statistical caution

Subject-level performance remains heterogeneous. Stable-48 / none provides the highest mean subject-level macro-F1 among the representative configurations and a smaller range than the full-feature and source-MI-only baselines, but the improvement is not uniform across participants.

Stable-48 / align further reduces dispersion but has lower complete-window macro-F1. Its paired comparison with the full-feature baseline is not statistically significant, with a mean subject-level macro-F1 difference of −0.0675, a 95% confidence interval from −0.1879 to 0.0748, and *p* = 0.293.

The subject-level evidence therefore supports cautious interpretation. Larger and more diverse target cohorts are needed before stronger claims of subject-level robustness or deployment generalizability can be made.

### 5.11 Reverse-direction and six-activity validation

#### 5.11.1 Matched reverse-direction transfer.

Table 15 reports the matched *PAMAP2* → *MHEALTH* transfer results obtained after reversing the source and target roles. Stable-48 achieved the highest complete-window accuracy, balanced accuracy, and macro-F1, with values of 0.5811, 0.5324, and 0.4400, respectively. The corresponding macro-F1 values were 0.4257 for Full-270 and 0.4320 for Source-MI-48. Thus, the aggregate macro-F1 advantage of Stable-48 over Full-270 and Source-MI-48 was 0.0143 and 0.0080, respectively.

**Table 15:** Matched reverse-direction and six-activity transfer results. All configurations use RF and no explicit target-feature transformation. Subject mean F1 is the unweighted mean of participant-level macro-F1 values, and $\overset{―}{S}$ is the mean descriptor-stability penalty.

Transfer setting	Configuration	Features	Acc.	Bal. acc.	Macro-F1	Subject F1	$\overset{―}{S}$
*PAMAP2* → *MHEALTH*, three-state	Full-270	270	0.5445	0.5145	0.4257	0.4221	0.2575
	Source-MI-48	48	0.5720	0.5257	0.4320	0.4320	0.3365
	Stable-48	48	**0.5811**	**0.5324**	**0.4400**	**0.4402**	**0.2571**
*MHEALTH* → *PAMAP2*, six-activity	Full-270	270	**0.4618**	**0.5055**	0.4077	**0.3743**	0.2882
	Source-MI-48	48	0.4237	0.4089	0.3162	0.2935	0.4054
	Stable-48	48	0.4459	0.4993	**0.4165**	0.3678	**0.2305**

The mean stability penalty of Stable-48 was 0.2571, compared with 0.3365 for Source-MI-48, representing a 23.6% reduction. The aggregate ordering therefore remained consistent after exchanging the source and target datasets, although the lower absolute scores indicate that the reverse direction is substantially more difficult under the evaluated protocol.

At the participant level, Stable-48 exceeded Full-270 for 9 of the 10 *MHEALTH* participants, with a mean paired macro-F1 difference of 0.0181 and a 95% bootstrap confidence interval of [−0.0185,0.0409]. Stable-48 exceeded Source-MI-48 for 8 participants, tied for one, and was lower for one, with a mean difference of 0.0082 and a 95% confidence interval of [0.0011, 0.0152]. The corresponding Holm-adjusted sign-flip *p*-values were 0.3320 and 0.1172. These participant-level contrasts therefore do not establish statistically significant superiority, despite the consistent aggregate ranking.

#### 5.11.2 Six-activity transfer.

Under the six-activity *MHEALTH* → *PAMAP2* evaluation, Stable-48 achieved the highest complete-window macro-F1 at 0.4165, compared with 0.4077 for Full-270 and 0.3162 for Source-MI-48. Full-270 retained slightly higher accuracy and balanced accuracy than Stable-48, with respective values of 0.4618 versus 0.4459 and 0.5055 versus 0.4993. Stable-48 therefore preserved the macro-F1 advantage of stability-aware selection, but did not dominate the full representation on every aggregate metric.

The mean selected-subset stability penalty was 0.2305 for Stable-48 and 0.4054 for Source-MI-48. Stability-aware selection consequently reduced the mean penalty by 43.1% under the finer-grained taxonomy while achieving a 0.1003 higher complete-window macro-F1 than source-only feature selection.

Participant-level macro-F1 was 0.3678 for Stable-48, 0.3743 for Full-270, and 0.2935 for Source-MI-48. The paired Stable-48 minus Full-270 difference was −0.0065, with a 95% confidence interval of [−0.0528,0.0377] and $p_{Holm}=0.7969$. The Stable-48 minus Source-MI-48 difference was 0.0743, with a 95% confidence interval of [0.0030, 0.1377] and $p_{Holm}=0.1875$. These results support improved aggregate macro-F1 and descriptor stability relative to Source-MI-48, while showing that the advantage over Full-270 is not uniform across participants.

Table 16 summarizes the paired participant-level macro-F1 contrasts for the reverse-direction and six-activity evaluations.

**Table 16:** Paired participant-level macro-F1 contrasts for the matched reverse-direction and six-activity evaluations. Confidence intervals are obtained by participant-level bootstrap resampling. Holm adjustment is applied to the two planned contrasts within each evaluation.

Evaluation	Contrast	Participants	Mean diff.	95% CI	Pos./tie/neg.	$p_{Holm}$
Reverse three-state	Stable-48 minus Full-270	10	0.0181	[−0.0185,0.0409]	9/0/1	0.3320
Reverse three-state	Stable-48 minus Source-MI-48	10	0.0082	[0.0011, 0.0152]	8/1/1	0.1172
Six-activity	Stable-48 minus Full-270	8	−0.0065	[−0.0528,0.0377]	4/0/4	0.7969
Six-activity	Stable-48 minus Source-MI-48	8	0.0743	[0.0030, 0.1377]	6/0/2	0.1875

Fig 8 compares the three matched feature representations across the complete-window metrics. Stable-48 achieves the highest values for all three metrics in the reverse direction and the highest macro-F1 in the six-activity evaluation.

**Fig 8:**
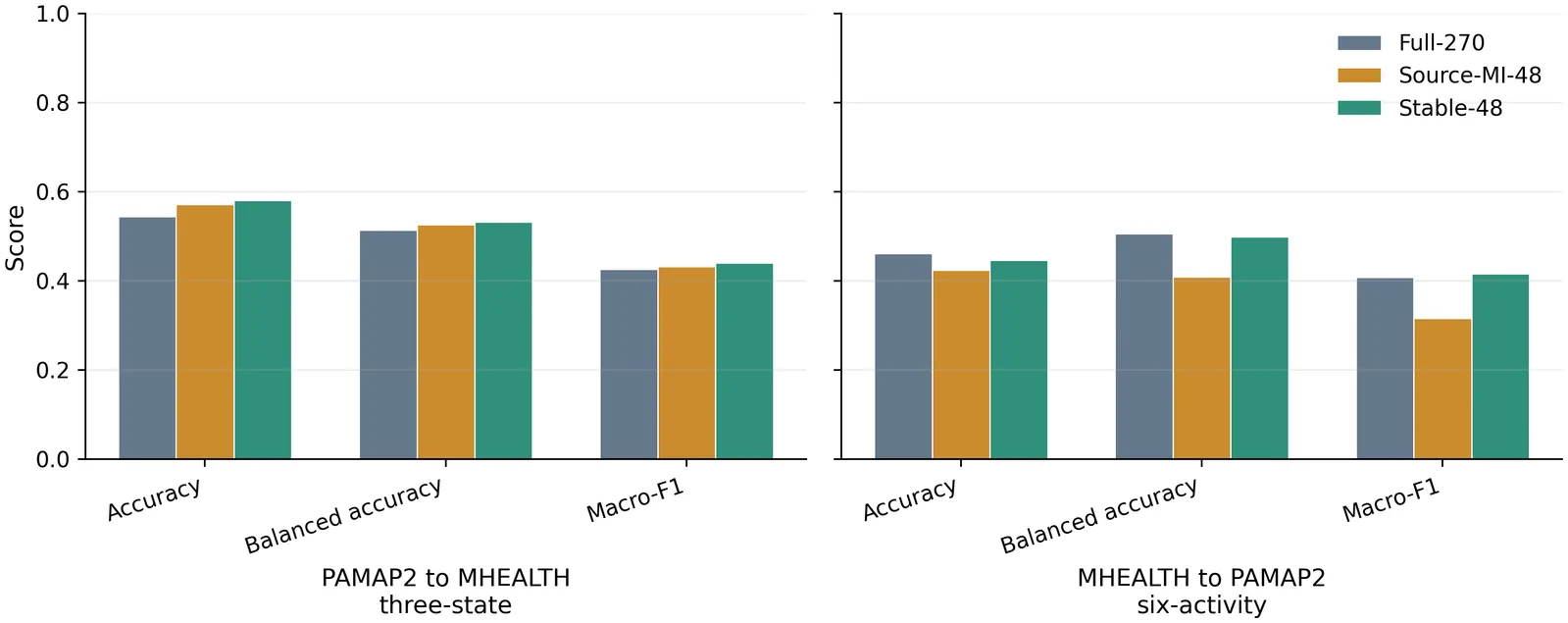
Complete-window performance of Full-270, Source-MI-48, and Stable-48 under reverse three-state *PAMAP2* → *MHEALTH* transfer and six-activity *MHEALTH* → *PAMAP2* transfer. All configurations use the same fixed RF settings and no explicit target-feature transformation.

Table 17 reports the class-wise Stable-48 results. Running achieved the highest F1-score at 0.8592, followed by lying at 0.6072. Sitting and stair climbing both achieved F1-scores of approximately 0.495. Standing and walking remained difficult to distinguish, with F1-scores of 0.0026 and 0.0397, respectively.

**Table 17:** Class-wise Stable-48 performance for the six-activity *MHEALTH* → *PAMAP2* evaluation.

Activity	Precision	Recall	F1-score	Support
Lying	0.9013	0.4578	0.6072	758
Sitting	0.3567	0.8093	0.4952	729
Standing	0.0313	0.0013	0.0026	748
Walking	0.1826	0.0222	0.0397	944
Stair climbing	0.3655	0.7676	0.4952	839
Running	0.7930	0.9375	0.8592	384

The normalized confusion matrix in Fig 9 shows that 89% of standing windows were predicted as sitting and 96% of walking windows were predicted as stair climbing. These confusions demonstrate the additional difficulty created when posture-dominated and locomotor activities are no longer pooled within the broader three-state taxonomy.

**Fig 9:**
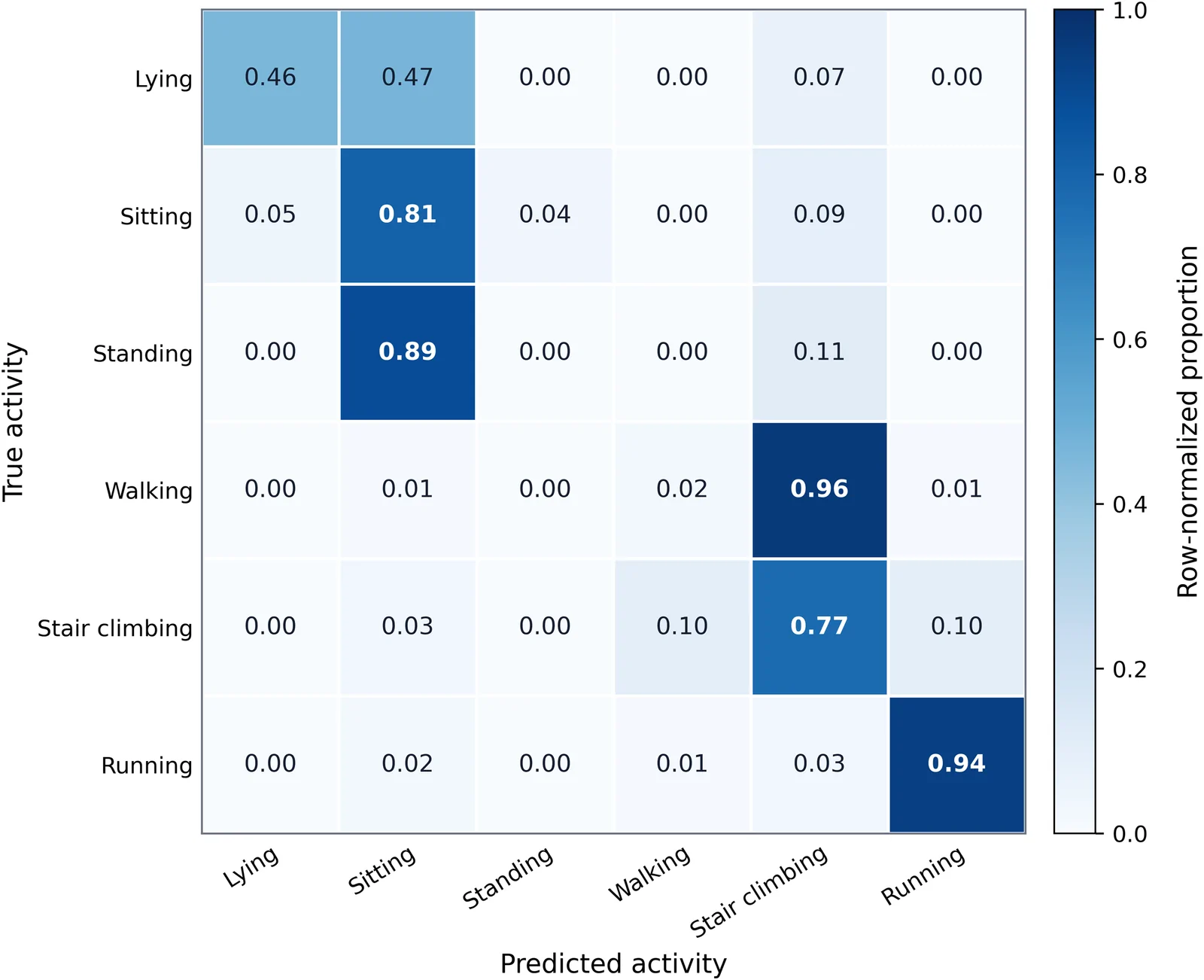
Row-normalized confusion matrix for Stable-48 under the six-activity *MHEALTH* → *PAMAP2* evaluation. Rows indicate true activities and columns indicate predicted activities.

#### 5.11.3 Exploratory multi-dataset transfer.

Table 18 shows pronounced transfer asymmetry across *MHEALTH*, *PAMAP2*, and *UCI HAR*. Under the unified exploratory protocol, *MHEALTH* → *PAMAP2* is the strongest pair, whereas *UCI HAR* → *PAMAP2* is the weakest.

**Table 18:** Exploratory cross-dataset transfer results under a unified evaluation protocol.

Source	Target	Target Macro-F1	Target Accuracy
*MHEALTH*	*PAMAP2*	0.7890	0.8426
*MHEALTH*	*UCI HAR*	0.6625	0.7806
*PAMAP2*	*MHEALTH*	0.7632	0.8081
*PAMAP2*	*UCI HAR*	0.5640	0.6747
*UCI HAR*	*MHEALTH*	0.6154	0.5741
*UCI HAR*	*PAMAP2*	0.4995	0.5302

These results are not directly comparable with the primary experiment or the matched evaluations in Table 15 because the exploratory protocol applies a common setting to datasets with different sensor configurations and task structures. In particular, the exploratory *PAMAP2* → *MHEALTH* row does not represent the matched fixed-parameter comparison among Full-270, Source-MI-48, and Stable-48. These exploratory results are therefore interpreted separately from the matched primary and reverse-direction evaluations.

## 6 Discussion

### 6.1 Principal findings

This study evaluated whether compact handcrafted descriptors remain useful when transferred from *MHEALTH* to the external *PAMAP2* dataset under differences in participants, sensor configuration, body placement, sampling rate, acquisition protocol, and activity composition. The external transfer experiment was more informative than the near-ceiling source-domain results because it directly exposed the effects of cross-dataset shift.

Under the pre-specified *MHEALTH* → *PAMAP2* protocol and primary three-state mapping, Stable-48 / none achieved an accuracy of 0.9042 and a macro-F1 of 0.8753, while reducing the representation from 270 to 48 descriptors. Its higher complete-window performance relative to both the full representation and the source-MI-only subset indicates that the improvement is associated with jointly considering source-domain relevance and unlabeled source–target stability rather than dimensionality reduction alone.

The reverse *PAMAP2* → *MHEALTH* evaluation preserved the same aggregate ordering under the fixed no-transformation configuration. Stable-48 achieved an accuracy of 0.5811 and a macro-F1 of 0.4400, compared with 0.5720 and 0.4320 for Source-MI-48 and 0.5445 and 0.4257 for Full-270. Stable-48 also had a 23.6% lower mean stability penalty than Source-MI-48. The lower absolute performance indicates substantial directional asymmetry under the evaluated protocol, while the repeated aggregate ordering indicates that the primary finding was not restricted to a single assignment of the two datasets as source and target.

The reverse-direction participant analysis provides a more qualified interpretation. Stable-48 exceeded Full-270 for 9 of 10 target participants and Source-MI-48 for 8 participants, with one tie in the latter comparison. However, the corresponding Holm-adjusted tests were not significant. The reverse evaluation therefore supports directional consistency in aggregate performance but does not establish universal or statistically confirmed participant-level superiority.

The six-activity evaluation examined whether the stability criterion remained useful after separating lying, sitting, standing, walking, stair climbing, and running. Stable-48 achieved the highest complete-window macro-F1 at 0.4165, compared with 0.4077 for Full-270 and 0.3162 for Source-MI-48. Its mean stability penalty was 0.2305, which was 43.1% lower than the 0.4054 obtained by Source-MI-48. Full-270 nevertheless retained slightly higher accuracy, balanced accuracy, and mean participant-level macro-F1 than Stable-48. The stability-aware subset therefore preserved a macro-F1 and descriptor-stability advantage, particularly over source-only selection, but did not dominate the full representation on every metric.

The six-class confusion structure also shows why the finer taxonomy is substantially more difficult. Running remained well recognized, whereas standing was predominantly classified as sitting and walking was predominantly classified as stair climbing. These errors indicate that descriptor stability alone cannot fully resolve biomechanically similar activities when their source and target sensor signatures differ. The three-state taxonomy is consequently best understood as a low-complexity monitoring abstraction that reduces incompatibilities between the original dataset taxonomies, not as a clinical classification system or a substitute for fine-grained activity recognition.

The parameter and component analyses qualify these findings. The primary values *K* = 48 and α=0.60 were fixed as methodological operating points rather than selected by maximizing target-domain performance. Although *K* = 24 achieved a higher post-hoc macro-F1, replacing the pre-specified setting on this basis would introduce target-guided selection. Similarly, the component ablation showed that mean shift and the rank-discrepancy term contributed differently, whereas the no-JSD variant performed better under the primary transfer direction. The complete score is retained because it was specified before final target-label evaluation; the *K* = 24 and no-JSD results are treated as post-hoc findings requiring independent validation.

The label-harmonization sensitivity analysis further showed that both the magnitude of the transfer results and the ordering of the tested configurations depend on how activities are assigned to the three states. The principal claims are therefore conditional on the pre-specified mapping and should not be interpreted as invariant to alternative biomechanical or intensity-based taxonomies. The six-activity results reinforce this qualification by showing that separating posture-dominated and locomotor activities changes both the selected descriptors and the resulting class-specific errors.

Finally, the primary setting is unsupervised with respect to target labels but target-aware with respect to unlabeled target-feature distributions. It is therefore not a strict zero-target-data deployment scenario. Because target windows are also clustered within a limited number of participants, the complete-window improvements in both transfer directions should be interpreted as descriptive transfer performance rather than definitive evidence of universal or subject-level superiority.

### 6.2 Role of stability-aware feature selection

The feature-count analysis indicates that compact settings between *K* = 24 and *K* = 48 remain competitive. The post-hoc *K* = 24 result suggests that more aggressive compression may be viable, but *K* = 48 remains the primary setting because it was fixed before final target-label evaluation, preserves descriptors from all five predefined families, and represents the intended moderate compactness operating point. Neither setting should be interpreted as universally optimal.

The relevance–stability analysis also favors an intermediate balance. Values of α=0.50 and α=0.60 produced similar transfer performance, whereas the strongly source-dominant α=0.90 setting performed substantially worse. This pattern suggests that source-domain relevance alone may retain descriptors that are discriminative within *MHEALTH* but less stable under the distributional differences encountered in *PAMAP2*.

The reverse-direction results extend this interpretation beyond the primary assignment of the two datasets. Under *PAMAP2* → *MHEALTH* transfer, Stable-48 reduced the mean selected-subset stability penalty from 0.3365 for Source-MI-48 to 0.2571, a reduction of 23.6%, while increasing complete-window macro-F1 from 0.4320 to 0.4400. Stable-48 also retained only 48 descriptors while attaining approximately the same mean stability penalty as Full-270, which obtained 0.2575.

The six-activity analysis produced a larger separation between the two compact subsets. Stable-48 reduced the mean stability penalty from 0.4054 for Source-MI-48 to 0.2305, corresponding to a 43.1% reduction, while increasing macro-F1 from 0.3162 to 0.4165. The lower penalty is expected from the feature-selection objective, but its co-occurrence with higher macro-F1 in both the reverse-direction and six-activity evaluations supports the intended role of the stability term in filtering source-relevant descriptors that undergo stronger marginal shift.

This relationship is not sufficient to guarantee superiority over the full representation for every outcome. In the six-activity evaluation, Full-270 retained slightly higher accuracy, balanced accuracy, and mean participant-level macro-F1 than Stable-48. The stability penalty is therefore best interpreted as a feature-level shift criterion that complements source relevance, rather than as a direct surrogate for target classification performance or participant-level robustness.

The feature-family analysis supports this interpretation. Correlation and frequency-domain descriptors were more stable on average, whereas statistical and temporal-dynamics descriptors showed stronger source-domain relevance but greater cross-domain variation. Relevance and stability are therefore complementary properties rather than interchangeable criteria.

The component ablation showed that normalized mean shift provided the clearest contribution to aggregate performance and subject-level consistency. The rank-discrepancy term produced a smaller effect and is best interpreted as a weak label-free regularizer rather than an estimate of target-domain class relevance. By contrast, JSD did not provide incremental benefit for the primary transfer direction. This may indicate that histogram-based marginal divergence penalized differences that did not necessarily reduce class separability. Because this ablation finding is based on the primary source–target direction, a simplified mean-shift–rank formulation should not be adopted without independent validation.

The mapping-sensitivity results introduce an additional qualification: changing the activity taxonomy modifies the source labels, source mutual information, selected descriptors, target class distribution, and class-specific errors. The six-activity evaluation confirms that the stability-aware subset itself changes when biomechanically similar activities are separated. Stability-aware selection should therefore be evaluated jointly with the adopted harmonization rather than assumed to be taxonomy invariant.

### 6.3 Comparison with feature-level neural and domain-adaptation baselines

Among the tested feature-level neural models, DANN-MLP achieved the highest accuracy and macro-F1, followed by Deep CORAL-MLP and Source-MLP. Within the selected handcrafted descriptor space, this ordering indicates that covariance-based and adversarial adaptation can improve transfer relative to a source-only MLP.

Stable-48 / none nevertheless achieved higher complete-window accuracy and macro-F1 than all three neural baselines under the common 48-dimensional representation and target-label-free protocol. This comparison is deliberately restricted to the implemented descriptor space. Source-MLP, Deep CORAL-MLP, and DANN-MLP do not learn directly from raw sensor windows and do not constitute exhaustive benchmarks of convolutional, recurrent, transformer-based, contrastive, self-supervised, or foundation-style time-series models. The results therefore do not establish general superiority over deep learning or neural domain adaptation for wearable activity recognition.

### 6.4 Main transfer configuration and deployment-balanced operation

Stable-48 / none is retained as the main transfer configuration because it provides the strongest complete-window accuracy and macro-F1 among the principal compact settings under the primary mapping. It therefore represents the accuracy-oriented operating point of the proposed framework.

Stable-48 / align provides a different trade-off. It combines compactness, high vigorous-state recall, lower subject-level dispersion, and low model-inference latency, but its macro-F1 is lower. It should therefore be described as a deployment-balanced operating point rather than as the best or statistically superior model.

The mapping-sensitivity results also show that this ordering is not universal. Stable-48 / align performed better than Stable-48 / none under both alternative mappings. The preferred configuration consequently depends on the activity taxonomy and on whether balanced classification or vigorous-state sensitivity is prioritized.

### 6.5 Reliability, calibration, and vigorous-state interpretation

The class-wise analysis shows that aggregate performance alone is insufficient for interpreting transfer reliability. Under the primary mapping, the deployment-balanced configuration achieves high vigorous-state recall but lower precision because some active windows are classified as vigorous. This behavior may be acceptable in sensitivity-oriented monitoring, but it may generate excessive false positives if isolated window-level outputs are treated as actionable alarms.

Vigorous-state behavior also depends strongly on the adopted harmonization. The alternative mappings changed both vigorous-state prevalence and recall, confirming that class-specific reliability cannot be interpreted independently of the underlying activity definition.

The six-activity evaluation provides a more demanding assessment of class-specific reliability. Stable-48 retained strong running performance, with a recall of 0.9375 and an F1-score of 0.8592, while lying, sitting, and stair climbing achieved F1-scores of 0.6072, 0.4952, and 0.4952, respectively. Standing and walking were substantially more difficult, with F1-scores of 0.0026 and 0.0397.

The confusion matrix shows that 89% of standing windows were classified as sitting and 96% of walking windows were classified as stair climbing. These errors occur between activities with related postural or locomotor signal characteristics and demonstrate that a favorable aggregate stability score does not ensure reliable separation of every activity pair. They also show that the three-state mapping can conceal specific within-state confusions when sitting and standing or walking and stair climbing are grouped into broader monitoring categories.

Consequently, the three-state output is suitable only as a coarse wearable-derived monitoring abstraction. Applications requiring reliable posture discrimination, gait characterization, stair detection, or activity-specific clinical interpretation would require validation under the fine-grained taxonomy and may benefit from additional placement-specific features, temporal context, independent calibration, or complementary sensor modalities.

The post-hoc threshold analysis illustrates the expected precision–recall trade-off but is not used for model selection or primary reporting. In a deployable system, decision thresholds should be pre-specified or calibrated using an independent validation set defined under the intended activity taxonomy. Temporal aggregation across consecutive windows may further reduce the effect of transient false-positive predictions.

### 6.6 Compactness, payload, and latency

Reducing the representation from 270 to 48 descriptors substantially lowers the estimated communication payload while preserving an interpretable input space. The *K* = 24 result suggests that further compression may be possible, but this setting requires independent validation before being recommended for deployment.

The communication analysis quantifies payload size only and should not be interpreted as measured energy savings. Similarly, the sub-0.03 ms latency corresponds to model inference after descriptor extraction rather than end-to-end execution. In the evaluated software implementation, feature extraction required approximately 30 ms per 5-s window and dominated the processing cost. Sensing, buffering, wireless transmission, device scheduling, and dashboard-side processing were not measured.

### 6.7 Subject-level heterogeneity and statistical caution

Complete-window metrics summarize all target predictions but may conceal inter-individual variability because several overlapping windows originate from each participant. The subject-level analysis confirmed heterogeneous performance across the retained *PAMAP2* subjects.

Stable-48 / none achieved a higher mean subject-level macro-F1 and a smaller range than the full-feature and source-MI-only baselines, but the improvement was not uniform across participants. The *K* = 24 configuration also showed a smaller post-hoc range, but this result cannot establish superior subject-level robustness because the setting was identified after target evaluation.

The reverse-direction evaluation showed a similar distinction between aggregate and participant-level evidence. Stable-48 achieved a mean participant-level macro-F1 of 0.4402, compared with 0.4320 for Source-MI-48 and 0.4221 for Full-270. Stable-48 exceeded Full-270 for 9 of 10 *MHEALTH* participants, but the mean paired difference of 0.0181 had a 95% bootstrap confidence interval of [−0.0185,0.0409] and $p_{Holm}=0.3320$. Relative to Source-MI-48, the mean difference was 0.0082, with 8 positive participant differences, one tie, one negative difference, and $p_{Holm}=0.1172$. The aggregate reverse-direction ordering is therefore encouraging but does not establish participant-level superiority.

Under the six-activity taxonomy, Stable-48 achieved the highest complete-window macro-F1 but not the highest unweighted mean participant-level macro-F1. The participant means were 0.3678 for Stable-48, 0.3743 for Full-270, and 0.2935 for Source-MI-48. The Stable-48 minus Full-270 paired difference was −0.0065, with a 95% confidence interval of [−0.0528,0.0377] and $p_{Holm}=0.7969$. Stable-48 exceeded Source-MI-48 by an average of 0.0743, with a 95% confidence interval of [0.0030, 0.1377], but the Holm-adjusted exact sign-flip result remained non-significant at $p_{Holm}=0.1875$.

The difference between complete-window and participant-mean rankings reflects the unequal number of retained windows per participant and the fact that aggregate metrics weight participants according to their window counts. Reporting both summaries is therefore necessary to distinguish overall target-window performance from consistency across individuals.

The deployment-balanced configuration reduced subject-level dispersion but did not achieve the highest complete-window macro-F1, and its paired comparison with the full-feature baseline was not statistically significant. Given the limited number of retained target subjects across the primary, reverse-direction, and six-activity evaluations, the evidence supports cautious descriptive interpretation rather than definitive claims of inter-individual superiority. Larger and more diverse target cohorts are required to assess whether the observed subject-level trends are reproducible.

### 6.8 Limitations and future work

Several limitations should be considered. First, the study is based on secondary analysis of public datasets and does not include prospective daily-life or clinical deployment. The outputs should therefore be interpreted as wearable-derived activity indicators rather than diagnostic decisions, clinical alarms, or substitutes for validated functional assessment instruments.

Second, the three-state harmonization necessarily simplifies the original activity taxonomies. Activities grouped within the same state may differ in intensity, posture, movement pattern, and biomechanical meaning, while similarly named activities may be performed or annotated differently across datasets. The sensitivity analysis confirmed that alternative mappings can change class prevalence, transfer performance, vigorous-state recall, and the ordering of the tested configurations. The lowest-motion state also includes standing and should therefore be interpreted as an operational stationary/postural category rather than consensus-defined sedentary behavior. The three states are not validated clinical, functional, or metabolic-intensity categories.

The six-activity evaluation reduced this pooling but revealed substantial fine-grained transfer difficulty. Stable-48 retained the highest complete-window macro-F1 and the lowest compact-subset stability penalty, whereas Full-270 achieved slightly higher accuracy, balanced accuracy, and mean participant-level macro-F1. Standing and walking were also frequently confused with sitting and stair climbing, respectively. The present results therefore do not establish uniformly reliable recognition across all six activities. Future studies should define harmonization rules using externally justified criteria, such as measured energy expenditure, metabolic-equivalent ranges, biomechanical similarity, or expert-defined application requirements, and should validate activity-specific performance in larger participant cohorts.

Third, the bidirectional validation remains restricted to *MHEALTH* and *PAMAP2*. Reversing their roles provides a distinct source–target direction and reproduces the aggregate ordering of Full-270, Source-MI-48, and Stable-48. However, both directions share the same two datasets, sensor families, and harmonization decisions. Matched validation using an additional dataset and the same frozen operating values is still needed to assess broader cross-dataset generalizability.

Fourth, the method uses unlabeled target-domain distributions for stability estimation and is therefore target-aware rather than strictly source-only. Future work should compare this setting with domain generalization, multi-source transfer, limited-target-data adaptation, and self-supervised learning under explicit safeguards against target-label leakage.

Fifth, the target-variance quantity in the rank-discrepancy term is only a label-free dispersion and non-degeneracy proxy. It does not estimate target-domain class relevance and may reflect noise, participant heterogeneity, or acquisition mismatch. Its contribution and interpretation should be evaluated across additional datasets and transfer directions.

Sixth, the stability weights, *K* = 48, and α=0.60 were pre-specified but are not claimed to be universally optimal. The post-hoc *K* = 24 and no-JSD results suggest potentially useful alternatives, but adopting either on the basis of the current target-domain results would constitute retrospective selection. Additional independent source–target pairs beyond the bidirectional *MHEALTH*–*PAMAP2* evaluation are needed before recommending a different feature count or simplified stability score.

Seventh, the vigorous-state threshold analysis is retrospective, and the deployment-balanced configuration retains limited vigorous-state precision. Future work should use independent threshold calibration and examine temporal aggregation of repeated activity predictions.

Eighth, the neural comparisons are restricted to feature-level Source-MLP, Deep CORAL-MLP, and DANN-MLP models. They do not represent strong or exhaustive raw-signal deep-learning baselines. Future comparisons should include convolutional, recurrent, transformer-based, contrastive, self-supervised, and foundation-style approaches under matched preprocessing, subject splits, target-label restrictions, and computational constraints.

Finally, deployment evidence is limited to software-side latency, model size, and estimated communication payload. Embedded evaluation is needed to measure end-to-end latency, memory demand, feature-extraction cost, wireless overhead, and energy consumption. The limited demographic and clinical metadata in the public datasets also prevent comprehensive assessment of subgroup robustness, fairness, and calibration. Future validation should therefore include larger and more diverse populations, richer metadata, different sensor placements, mobility limitations, and longitudinal monitoring scenarios.

## 7 Conclusion

This study evaluated compact stability-aware feature selection for cross-dataset wearable activity monitoring. The proposed framework combines handcrafted wearable-signal descriptors, unlabeled source–target descriptor-stability analysis, compact feature selection, and lightweight classification. In the primary three-state setting, its outputs are coarse wearable-derived indicators sedentary, active, and vigorous rather than diagnostic decisions, clinical alarms, or validated clinical assessments. These labels operationally represent stationary or posture-dominated behavior, routine functional movement, and high-dynamic movement, respectively. Because the lowest-motion state includes standing, its *sedentary* label should not be interpreted as consensus-defined sedentary behavior.

Under the pre-specified *MHEALTH* → *PAMAP2* protocol and primary three-state mapping, Stable-48 / none achieved an accuracy of 0.9042 and a macro-F1 of 0.8753, while reducing the representation from 270 to 48 descriptors. It achieved higher complete-window accuracy and macro-F1 than the full-feature baseline, the source-MI-only compact subset, the evaluated shallow target-feature-transformation configurations, and the tested feature-level neural adaptation baselines within the common handcrafted descriptor space. These findings indicate that combining source-domain discriminative relevance with unlabeled source–target descriptor stability can improve descriptive target-domain performance while substantially reducing dimensionality.

The reverse *PAMAP2* → *MHEALTH* evaluation reproduced the same aggregate ordering using the frozen no-transformation configuration. Stable-48 achieved an accuracy of 0.5811 and a macro-F1 of 0.4400, compared with 0.5720 and 0.4320 for Source-MI-48 and 0.5445 and 0.4257 for Full-270. Stable-48 also reduced the mean compact-subset stability penalty by 23.6% relative to Source-MI-48. The participant-level contrasts were not statistically significant after Holm adjustment. Accordingly, the reverse evaluation supports consistency of the aggregate ranking across both transfer directions but does not establish universal or participant-level superiority.

The primary configuration is unsupervised with respect to target labels but target-aware with respect to unlabeled target-feature distributions. It is therefore not a strict zero-target-data generalization setting. Harmonized target labels are accessed only after prediction generation for final evaluation; original activity identifiers are used only to apply the pre-specified activity-inclusion and window-boundary rules. The pre-specified values *K* = 48 and α=0.60 are retained as methodological operating points rather than as universally optimal choices. Although the post-hoc *K* = 24 configuration achieved a higher macro-F1, adopting it on the basis of the current target-domain results would constitute retrospective target-guided selection. This alternative operating point therefore requires independent validation.

The component ablation showed that the three stability terms contributed unequally. Mean shift provided the clearest incremental benefit, the rank-discrepancy term had a smaller regularizing role, and the no-JSD variant performed better under the primary transfer direction. The complete score is nevertheless retained as the primary formulation because it was pre-specified, whereas the no-JSD result is treated as a post-hoc diagnostic finding. Additional independent source–target pairs are needed before adopting a simplified stability formulation.

The mapping-sensitivity analysis further showed that both transfer performance and configuration ordering depend on the adopted three-state harmonization. Stable-48 / none achieved the highest macro-F1 among the reported compact settings under the primary mapping, whereas Stable-48 / align achieved the highest macro-F1 under both alternative mappings. The principal findings are therefore conditional on the pre-specified taxonomy and should not be interpreted as invariant to alternative biomechanical or intensity-based harmonizations.

Under the finer-grained six-activity taxonomy, Stable-48 achieved the highest complete-window macro-F1 at 0.4165, compared with 0.4077 for Full-270 and 0.3162 for Source-MI-48. Its mean stability penalty was 43.1% lower than that of Source-MI-48. Full-270 nevertheless retained slightly higher accuracy, balanced accuracy, and mean participant-level macro-F1, while standing and walking remained difficult to separate from sitting and stair climbing. Stability-aware selection therefore retained a macro-F1 and descriptor-stability advantage under the finer taxonomy, particularly relative to source-only selection, but did not dominate every metric or resolve all biomechanically similar activities.

Overall, the results support stability-aware compact feature selection as a transparent and lightweight strategy for cross-dataset wearable activity monitoring under the evaluated protocols. The bidirectional results strengthen the evidence that the aggregate advantage is not confined to one source–target assignment, and the six-activity results show that the stability criterion remains informative when the taxonomy is refined. However, substantial activity-specific and subject-level variability remains, and the evidence does not establish universal robustness, clinical readiness, or general superiority over raw-signal deep-learning methods. Future work should evaluate additional independent source–target pairs, externally justified activity mappings, larger and more diverse cohorts, stronger raw-signal neural and domain-adaptation baselines, prospective daily-life monitoring, independent threshold calibration, temporal aggregation, subgroup robustness, and embedded implementation on wearable or edge devices.

## Supporting information

S1 FileMain reproducibility outputs.ZIP archive containing cross-dataset transfer results, source-domain benchmark results, target-subject performance summaries, feature-stability and feature-family summaries, bootstrap and statistical-comparison outputs, feature-selection and stability-component ablations, stability-weight and α-sensitivity results, label-harmonization sensitivity results, model trade-off analyses, modality-robustness results, deployment-balanced subject-level comparisons, and summary_results.json.(ZIP)

S2 File Neural-baseline outputs. ZIP archive containing results for Source-MLP, Deep CORAL-MLP, and DANN-MLP, including summary results, seed-specific confusion matrices and subject-level metrics, the selected-feature list, and the feature-stability inputs used in the neural-baseline analysis.
(ZIP)
